# Patterns of genomic and phenomic diversity in wine and table grapes

**DOI:** 10.1038/hortres.2017.35

**Published:** 2017-08-02

**Authors:** Zoë Migicovsky, Jason Sawler, Kyle M Gardner, Mallikarjuna K Aradhya, Bernard H Prins, Heidi R Schwaninger, Carlos D Bustamante, Edward S Buckler, Gan-Yuan Zhong, Patrick J Brown, Sean Myles

**Affiliations:** 1 Department of Plant, Food and Environmental Sciences, Faculty of Agriculture, Dalhousie University, Truro, NS B2N 5E3, Canada; 2 Anandia Labs, Vancouver, BC V6T 1Z4, Canada; 3 Agriculture and Agri-Food Canada, Fredericton Research and Development Centre, Fredericton, NB, Canada E3B 4Z7; 4 National Clonal Germplasm Repository, United States Department of Agriculture-Agricultural Research Service, University of California, Davis, CA 95616, USA; 5 United States Department of Agriculture, Agricultural Research Service, Grape Genetics Research Unit, New York State Agricultural Experiment Station, Cornell University, Geneva, NY 14456, USA; 6 Department of Genetics, Stanford University, Stanford, CA 94305, USA; 7 Department of Plant Breeding and Genetics, Cornell University, Ithaca, NY 14853, USA; 8 United States Department of Agriculture, Agricultural Research Service, Plant Genetic Resources Unit, New York State Agricultural Experiment Station, Cornell University, Geneva, NY 14456, USA; 9 Department of Crop Science, University of Illinois, Urbana, IL 61801, USA

## Abstract

Grapes are one of the most economically and culturally important crops worldwide, and they have been bred for both winemaking and fresh consumption. Here we evaluate patterns of diversity across 33 phenotypes collected over a 17-year period from 580 table and wine grape accessions that belong to one of the world’s largest grape gene banks, the grape germplasm collection of the United States Department of Agriculture. We find that phenological events throughout the growing season are correlated, and quantify the marked difference in size between table and wine grapes. By pairing publicly available historical phenotype data with genome-wide polymorphism data, we identify large effect loci controlling traits that have been targeted during domestication and breeding, including hermaphroditism, lighter skin pigmentation and muscat aroma. Breeding for larger berries in table grapes was traditionally concentrated in geographic regions where Islam predominates and alcohol was prohibited, whereas wine grapes retained the ancestral smaller size that is more desirable for winemaking in predominantly Christian regions. We uncover a novel locus with a suggestive association with berry size that harbors a signature of positive selection for larger berries. Our results suggest that religious rules concerning alcohol consumption have had a marked impact on patterns of phenomic and genomic diversity in grapes.

## Introduction

Grapes (*Vitis vinifera* L.), one of the first domesticated perennials, originated in the Near East 5000–8000 years ago^1^ and remain an economically and culturally important crop. In 2015, grapevines covered 7.5 million hectares and produced 76 million tons of grapes globally.^2^ Over the past millennia, human selection for traits of interest, especially those important to fruit production, have shaped the appearance of grapes. In particular, selection for hermaphroditic flowers increased grape production, as propagating both male and female plants was no longer required. While nearly half of all grapes grown are vinified into wine, 36% are consumed fresh and the rest are dried or used for juice.^2^ Desirable berry traits differ depending on the use of the grapes, and, thus, the different breeding targets for table and wine grapes have led to differences in berry and bunch size.^3,4,5^ There is also evidence of selection for white berry color.^5^

While grape breeding has resulted in selection for several traits over the past millennia, current consumer preference is focused on a small number of elite cultivars. As a result, most grape cultivars have been grown for centuries—such as ‘Pinot Noir’, which has existed for more than a millennium—using vegetative propagation. These genetically frozen cultivars are highly susceptible to continually evolving pathogens.^6,7^ Selection for new traits, including disease resistance, is a slow and expensive process in grapes. Breeding of new grape cultivars is hindered by high inbreeding depression as well as a lengthy juvenile phase lasting 3–5 years. Even after fruit production, additional time may be required to assess traits important for wine production.^4,8^ Developing a new grape cultivar using traditional breeding techniques takes 25–30 years. Fortunately, using genetic markers linked to phenotypes of interest can decrease the time required to develop new cultivars by up to 10 years.^8^ In addition, recent work estimated that use of marker-assisted selection (MAS) in grapes offered a cost-saving of 16–34%.^9^

The ability to save time and money when breeding makes grapes an attractive candidate for MAS.^7,10^ Using genetic markers, individuals can be tested for a trait at the seed or seedling stage. Thus, MAS offers the greatest potential for traits that are difficult and expensive to phenotype, such as disease resistance, or time-consuming to measure, such as fruit traits only visible after several years.^8^ Wild *Vitis* relatives have been previously used for hybrid grape breeding^11^ and are a promising source of resistance loci for introgression through MAS.^12^ For example, *V. arizonica* was used in the development of Pierce’s disease-resistant wine grapes,^13^ while *Muscadinia rotundifolia* was used to pyramid resistance from both powdery and downy mildew into *V. vinifera*.^14^ Markers have also been identified for many other traits in grape including berry color,^15,16^ flower sex,^17^ seedlessness^18^ and muscat aroma.^19^

The discovery of markers for agriculturally important traits has facilitated the use of MAS in grapes; however, the technique is only worthwhile when the cost of phenotyping is higher than the cost of discovering new markers and genotyping cultivars.^20^ Decreasing DNA sequencing costs will continue to accelerate both marker discovery and the implementation of MAS in grape breeding. While sequencing costs have decreased, phenotyping remains a slow and expensive process.^21^ Fortunately, historical phenotype information already available in gene banks can be linked with genomic information for genetic mapping of important traits. The ability to leverage historical data from gene banks for genetic mapping has previously been demonstrated in potato,^22^ barley^23^ and apple.^24^ Similarly, in grape, years of phenotype information may already be available and exploitable for the purposes of genetic mapping. Unfortunately, standardized data formatting and annotation are not yet widely adopted in grapevine and remain an essential goal.^25^

To investigate the history of selection in grape, as well as the future potential of MAS, we evaluated associations between 33 phenotypes and 6114 genome-wide single-nucleotide polymorphisms (SNPs) in 580 *V. vinifera* accessions from the United States Department of Agriculture (USDA) grape germplasm collection. We report several significant genome-wide association study (GWAS) results, demonstrate the use of signatures of selection as complementary to GWAS and find that phenotype relationships as well as patterns of genetic variation have been shaped by human culture and geography.

## Materials and methods

### Sample collection and genotype calling

The sample collection and genotype calling for the accessions used in this study were the result of previous work described in Myles *et al.*^26^ Briefly, samples were collected from the USDA grape germplasm collections in Davis, CA, and Geneva, NY. DNA was extracted using commercial extraction kits. A custom Illumina Vitis9KSNP array (San Diego, CA, USA) assaying 8898 SNPs was used to generate genotype data.^27^ Following quality filters (GenTrain Score ⩾0.3 and GenCall ⩾0.2), 6114 SNPs with <20% missing data in 1817 *Vitis* samples remained for analysis.^26^

### Data management

Phenotype data were downloaded from the USDA Germplasm Resources Information Network (GRIN; http://www.ars-grin.gov). Only accessions reliably identified as *V. vinifera* in Myles *et al.*^26^ were included. Measurements for flower sex were combined across years and samples with discordant values for flower sex across years were removed. Additional phenotype data including skin color, berry length, berry width, berry size and cluster density were collected as part of the present study. Our cluster density measures were merged with measurements available from GRIN, and when discrepancies between measurements existed, those values were removed. In some cases, phenotype data were recoded to facilitate genetic mapping. A complete description of the phenotype data, including recoding procedures, is available in Supplementary Table S1.

Phenotype data were only included in downstream analyses if measurements existed for at least 100 accessions, resulting in a final set of 33 unique phenotypes. While 2 years of data were available for four phenotypes, the correlation of trait values between years was often poor. The correlation between years was estimated using Pearson’s correlation for binary and quantitative phenotypes and Kendall’s rank correlation for ordinal phenotypes (Supplementary Table S2). For clarity, when 2 years of data were available for a given phenotype, data from the year with the greater sample size were included in the main portion of the manuscript. However, results for each year are presented separately in the Supplementary Material.

Pairs of accessions were considered to have a clonal relationship if $\hat{\pi }$ (proportion identity-by-descent), calculated using PLINK,^28^ exceeded 0.95. To avoid pseudoreplication, for each phenotype only the accession from a clonal group with the least amount of missing genotype data was included in downstream analyses. However, the accession’s phenotype was calculated as the average across all accessions within its clonal group. A Box–Cox transformation was applied to quantitatively measured traits when the distribution of observed values differed significantly from normality. The untransformed and transformed distributions for each phenotype are shown in Supplementary Figure S1. The phenotype distribution for ordinal traits is shown in Supplementary Figure S2. For binary traits, the majority phenotype was used instead of the mean when combining clones, and the distributions of these phenotypes are shown in Supplementary Figure S3. After all filtering steps, the final data set consisted of 33 phenotypes scored across 580 accessions and genotyped for 6114 SNPs.

### Patterns of phenotypic diversity

The correlations (*r*) between all pairwise phenotype comparisons were computed using R v3.2.0.^29^ Correlations between binary/binary, quantitative/quantitative and quantitative/binary phenotype pairs were tested using Pearson’s correlation. Correlations between quantitative/ordinal and binary/ordinal phenotype pairs were tested using Spearman's rank correlation coefficient. Finally, correlations between ordinal/ordinal phenotype pairs were tested using Kendall’s rank correlation. To correct for multiple comparisons, a Bonferroni correction was applied by multiplying *P*-values by the number of pairwise comparisons (528).

Accessions were divided based on use (table and wine) as well as geographic origin (East, Central and West; Supplementary Table S3). The East geographic region includes the Middle East as well as Russia, while the Central region includes Eastern Europe including Serbia, Hungary and Greece. Finally, the West region includes Western Europe such as France, Italy and Germany. A full list of the geographic origin of *V. vinifera* accessions in the USDA collection can be found in Myles *et al.*^26^ For each phenotype, we tested whether accessions with different uses and geography differed. We used a Fisher’s Exact test for binary phenotypes, a Mann–Whitney *U*-test for ordinal phenotypes and quantitative phenotypes. For the Fisher’s Exact test, we report the odds ratios. For the Mann–Whitney *U-*test, we report the *W-*test statistic. *P*-values were Bonferroni-corrected for multiple comparisons and all analyses were performed in R.

### Genetic population structure

Before assessing population structure, the genotype data were pruned for linkage-disequilibrium (LD) using PLINK by considering a window of 10 SNPs, removing 1 of a pair of SNPs if LD>0.5, and then shifting the window by 3 SNPs and repeating the procedure. Principal component analysis was performed on the resulting 3196 SNPs genotyped in 580 accessions using the smartpca program in the EIGENSOFT package.^30,31^ To investigate the degree to which population structure accounts for phenotypic variance within *V. vinifera*, we conducted linear regression for continuous and ordinal phenotypes, and logistic regression for binary phenotypes using trait values as response variables and eigenvalues for the first 10 principal components (PCs) as predictors. McFadden’s pseudo-*R*^2^ was calculated for logistic regression using the ‘pscl’ package^32^ in R v3.0.1. We define the phenotypic variance explained as the *R*^2^ of these models, for PC1, PC2 and PCs 3–10.

### Genomic prediction

To perform genomic prediction, SNPs with a MAF threshold of <0.01 were removed using PLINK,^28^ resulting in 4602 SNPs genotyped in 580 accessions. Missing genotype data were then imputed using LinkImpute^33^ with optimized values of 7 for parameter *k* and 23 for *l*, resulting in an estimated accuracy of 88.8%. Genomic prediction was performed on all phenotypes using imputed data and the x.val function in the R package PopVar.^34^ We selected the rrBLUP model and assessed prediction accuracy with a fivefold (nFold=5) cross-validation procedure repeated three times (nFold.reps=3) and no further filtering (min.maf=0). All other default parameters were used. The seedlessness phenotype was removed for this analysis because of an uneven binary trait distribution, which did not allow for cross-validation. Genomic prediction accuracy was calculated as the correlation (*r*) between the predicted phenotypes and the observed values.

### GWAS and selection scans

GWAS were performed using a linear mixed-model method, efficient mixed-model association expedited (EMMAX)^35^ and an identity-by-state kinship matrix calculated using the default EMMAX settings. The number of accessions included for each GWAS varied based on the phenotype, and, thus, a minor allele frequency (MAF) threshold of 0.01 was applied for each GWAS individually, resulting in 4409–4680 SNPs.

Haplotypes were inferred using fastPHASE^36^ as in Myles *et al.*^26^ SNPs with a MAF>0.05 and <10% missing data were included, resulting in 3397 SNPs. To identify potential regions of the *V. vinfera* genome under selection during domestication and breeding, we calculated the xpEHH statistic across the genome using selscan software.^37^ The xpEHH statistic requires the user to divide the samples into two groups to identify regions of the genome with unusually long stretches of low haplotype diversity in one group compared with the other. As this pattern of haplotype diversity is expected in regions subjected to positive selection, the identified regions are considered candidate regions that may harbor functional variants selected for during domestication or breeding. For berry size, we compared accessions with large berries (that is, within the top 10% of the berry size distribution) to accessions with small berries (that is, within the bottom 10% of the berry size distribution). For skin color, we compared dark-skinned accessions (that is, those with scores of 1 and 2) to light-skinned accessions (that is, those with scores of 4 and 5). We also compared groups divided based on several binary traits: Muscat versus non-Muscat, dioecious accessions versus hermaphrodites, and table versus wine grapes.

## Results and discussion

### Correlations between phenotypes

**Figure 1:**
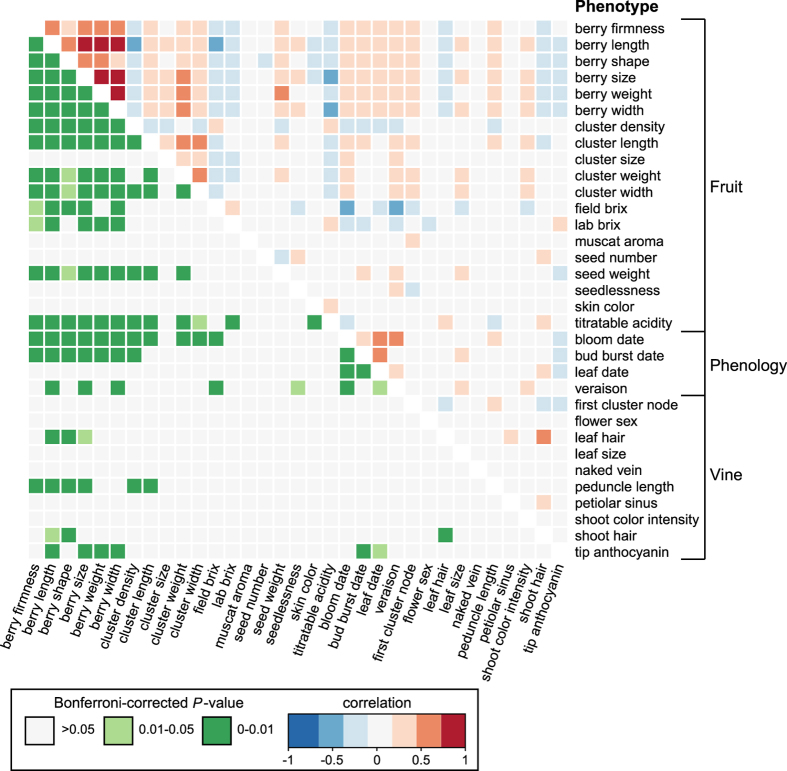
Correlations among grape phenotypes. Correlations were calculated using Pearson’s, Spearman’s or Kendall’s correlations depending on phenotypes compared (see Materials and methods). Values above the diagonal are colored to indicate the correlation results (*r*) and those below the diagonal indicate Bonferroni-corrected *P*-values.

We analyzed each of the 33 phenotypes in this study to uncover potential relationships between phenotypes, as well as confirm the reliability of the data. A correlation matrix between all phenotypes is shown in Figure 1.

We found that all measurements describing berry size were significantly correlated, including berry length and width (*r*=0.89, *P*<1×10^−15^) and berry size and weight (*r*=0.79, *P*<1×10^−15^). Thus, berries that are large according to one measure, such as length, also tend to be larger in other measures, such as width. In addition, all berry size measurements were positively correlated with berry firmness (for example, size: *r*=0.54, *P*<1×10^−15^, weight: *r*=0.48, *P*<1×10^−15^). Both large size and berry firmness are desirable traits in table grapes,^38^ and may have both been targeted by table grape breeders.

In addition to larger berries being firmer, we found that all berry size measurements were negatively correlated with cluster density including length (*r*=−0.43, *P*<1×10^−15^) and size (*r*=−0.41, *P*<1×10^−15^), indicating that larger berries were found in less dense clusters. This observation may have arisen from the divergent breeding targets of table and wine grape breeders: less dense clusters of large grapes are preferred in table grapes and smaller more densely packed berries are preferred in wine grapes.^38^ As expected, larger berries also tend to be found on larger clusters.

Titratable acidity, or the concentration of tartaric acid, was negatively correlated with berry size measurements including length (*r*=−0.42, *P*<1×10^−15^) and width (*r*=−0.44, *P*<1×10^−15^). Tartrate synthesis stops at veraison, the onset of ripening; therefore, an increase in berry size post veraison dilutes the amount of tartaric acid in the berry.^39^ Similarly, larger berries also tend to have a lower concentration of sugar.^40^ We found a negative correlation between lab brix and berry size (*r*=−0.32, *P*=6.50×10^−9^), as well as all other berry size measurements, providing further evidence that sugar concentration decreases as berry size increases.

Several phenological events, describing the timing of development, were assessed by various researchers and deposited into the USDA-GRIN database. Despite the noise expected from data collection across multiple years by multiple observers, many phenology traits are correlated with each other. For example, bud burst date and leaf date (*r*=0.44, *P*<1×10^−15^), leaf date and bloom date (*r*=0.61, *P*<1×10^−15^), bloom date and veraison (*r*=0.45, *P*=2.74×10^−7^) are all significantly correlated. Thus, the timing of a grapevine’s escape from dormancy is predictive of subsequent developmental milestones, like bud break, leaf production and the onset of veraison. This observation suggests that the genetic control of grapevine phenology may be at least partially coordinated by a single regulatory mechanism, rather than independent mechanisms for each developmental event.

### Phenotypic variance based on use and origin

**Figure 2:**
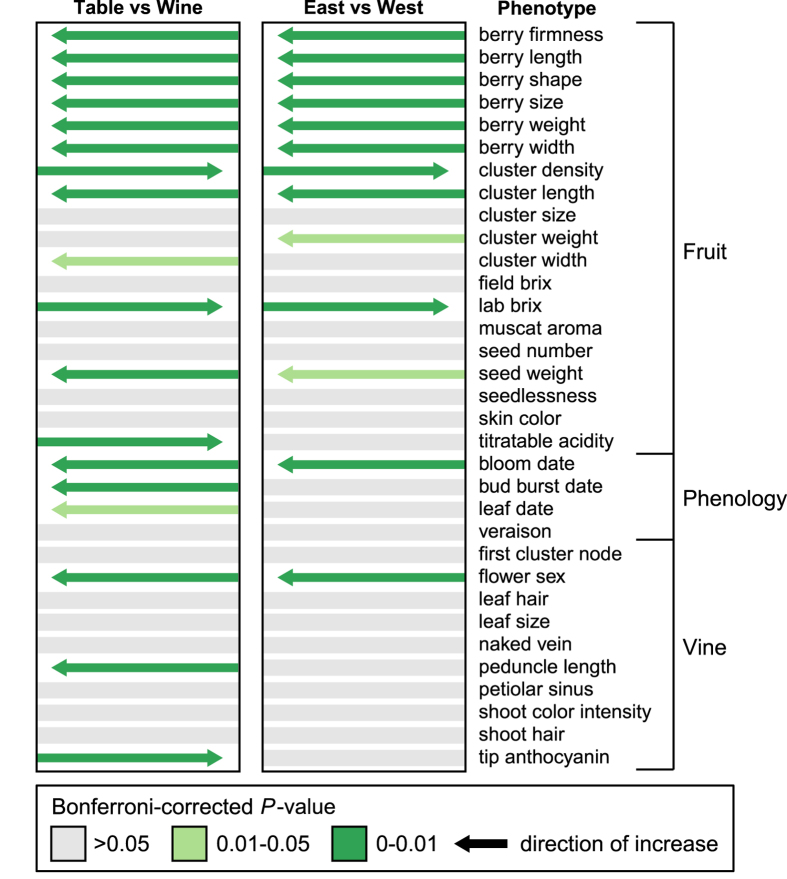
Relationship between grape phenotype and use or origin. Each phenotype was divided into two groups according use (table or wine) and origin (East or West) and compared. Significant increases are indicated by the direction of the arrow. *P*-values are Bonferroni-corrected.

In addition to the relationships between phenotypes, we examined differences between accessions based on use (table and wine) and geographic origin (East and West; Figure 2). Unlike wine grapes, which are pressed and fermented prior to consumption, table grapes are consumed directly and their desirability relies heavily on a visual assessment by the consumer. As a result, most well-known table grapes have large berries.^38^ Our results confirm that table grapes generally have berries that are greater in length (*W*=41 804.5, *P*<1×10^−15^), width (*W*=39 407.5, *P*<1×10^−15^), size (*W*=41 150, *P*<1×10^−15^) and weight (*W*=38 921.5, *P*<1×10^−15^) when compared with wine grapes. Smaller berries may be beneficial for winemaking, as smaller berries often have more desirable characteristics for vinification, including higher sugar concentration.^40,41^ Consistent with previous work, we also found that titratable acidity (*W*=23 250.5, *P*=2.67×10^−8^) and sugar content (*W*=23 792, *P*=5.09×10^−8^) were significantly higher in wine grapes.^42–45^

In addition to berry size, cluster density is important for table grapes, as very compact clusters are often damaged during packing and transport. Broken berries leak juice, which may spoil the entire cluster. A firm pulp texture that is not easily broken is therefore essential for table grapes.^38^ Table grapes were significantly firmer (*W*=45218, *P*=2.04×10^−14^) and had significantly less dense clusters (*W*=18006, *P*<1×10^−15^) than wine grapes, indicating that selective breeding likely created a divergence in these traits between table and wine grapes.

We also compared each phenotype for grapes originating in the East, primarily the Middle East, to those accessions originating in the West, primarily Western Europe (Figure 2b). Grapes from the East were significantly firmer (*W*=10564.5, *P*=4.65×10^−8^) and larger in size including length (*W*=9004, *P*=4.01×10^−9^), width (*W*=8832.5, *P*=3.14×10^−8^) and weight (*W*=8505, *P*=5.05×10^−8^). Eastern accessions also had less dense (*W*=4364, *P*=3.62×10^−8^), longer (*W*=10502.5, *P*=1.96×10^−7^) and heavier (*W*=7014, *P*=2.80×10^−4^) clusters when compared with the West.

**Figure 3:**
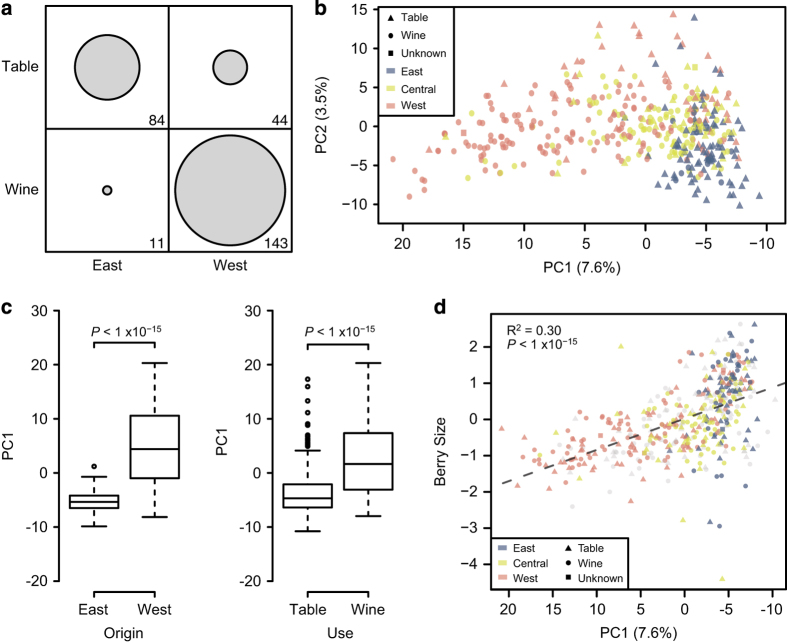
Genetic structure based on use, geographic origin, berry size and berry color. Principal component analysis (PCA) was performed using genome-wide SNP data. The percentage of the variance explained by each PC is indicated in parentheses along each axis. (**a**) In all, 282 accessions had information for both geographic origin and use. Circled areas are proportional to the number of observations within each category. Plot was created using the R package tableplot.^66^ (**b**) Accessions are labeled according to use with point shape (table, wine or unknown) as well as geographic origin in Europe based on point color (East, Central or West). PCs were determined using all accessions, but only those with geographic information are shown. (**c**) Boxplot of PC1 values for East and West as well as wine and table grapes. Results are reported from a Mann–Whitney *U*-test. (**d**) Correlation (*r*^2^) between PC1 and berry size. Accessions are labeled according to use (point shape) and geography (point color). Accessions without use or geography information are colored in gray. *R*^2^ and *P*-value are reported from a Pearson correlation test. The PC1 axis is shown in reverse order to be consistent with geography (that is, East to the right and West to the left).

The similarities between phenotype comparisons based on use and geography are expected, given that most table grapes are from the East and most wine grapes are from the West. In our data set there are 282 accessions with use, geography and phenotype information: 30% are Eastern table grapes but only 16% are Western table grapes. In comparison, 51% of the accessions are Western wine grapes and only 4% are Eastern wine grapes (Figure 3a). The paucity of Eastern wine grapes observed here is likely driven by religion. Islam is the dominant religion in the Eastern geographic area defined here and the consumption of alcohol has been prohibited among Muslims for over a millennium. Grape breeding in the East has therefore focused on the development of table grapes with desirable traits like large berry and bunch size.^4,6^ Conversely, as Christianity does not prohibit alcohol consumption and it has been the dominant religion in Western Europe, grapes from the West have generally been selected for their ability to produce high-quality wine. Thus, an analysis of the phenotype data alone reveals the strong influence of religion on shaping global patterns of grape phenotypic diversity.

### Genetic structure and genomic prediction

We investigated the genetic structure of grape accessions by performing principal component analysis using genome-wide SNP data. Accessions were labeled according to use (table or wine) and origin (East, Central or West) and plotted along PC1 and PC2 (Figure 3b). The primary axis of genetic structure (PC1) distinguished grapes from the East and West (*W*=1819, *P*<1×10^−15^) as well as table and wine grapes (*W*=17019, *P*<1×10^−15^; Figure 3c). Such grouping according to use and geography was also found in previous work that examined 2000 grape accessions from 52 countries.^3^ Given that most table grapes are from the East and most wine grapes are from the West (Figure 3a), it is not surprising to find similar population structure differences when comparing accessions based on geography and use. In addition, we found a significant relationship between berry size and PC1* *(*r*^2^=0.30, *P*<1×10^−15^; Figure 3d). Table grapes have significantly larger berries than wine grapes (Figure 2), and the strong selection by table grape breeders for large size has likely been a significant factor in the genetic differentiation between table and wine grapes.

**Figure 4:**
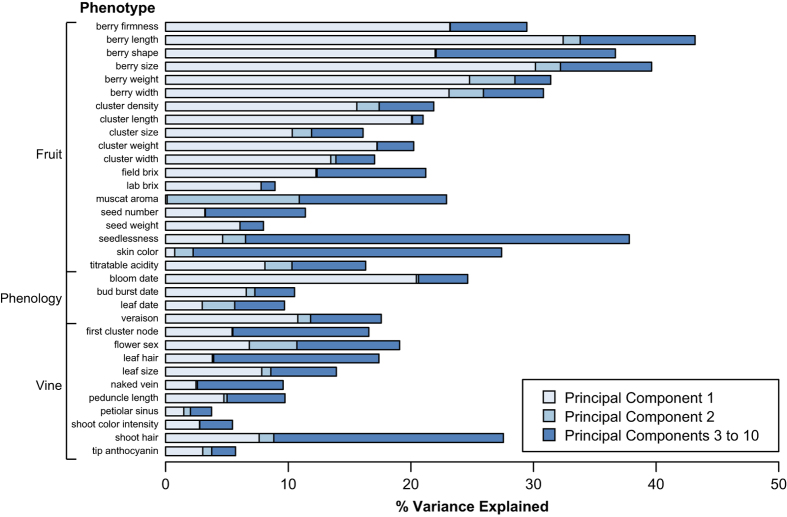
Proportion of variance explained for each phenotype using PCs 1–10. PCs were calculated using genome-wide SNPs.

Phenotypes that are strongly correlated with population structure are more likely to have been targeted by selection. Moreover, as population structure is a confounding effect in GWAS, phenotypes strongly correlated with population structure can be problematic to map using association mapping. We therefore examined the degree to which each phenotype is correlated with population structure. We found that the proportion of the phenotypic variance explained by genetic PCs 1 through 10 ranged from 2 to 43% across phenotypes (Figure 4). Most notably, PC1 explained a large proportion of the variance for berry shape and size measurements. This relationship is expected, given that PC1 is significantly correlated with berry size (Figure 3d) and all berry size and shape measurements are significantly correlated with each other (Figure 1). These observations suggest that selection for table grapes in the East and wine grapes in the West has resulted in berry size being strongly correlated with the overall genetic structure of grapes.

In addition to berry traits, the only other phenotype for which the first 10 genotypic PCs explain over 30% of the phenotypic variance is seedlessness (38%). In contrast to berry phenotypes, only a small proportion of the variance in seedlessness is explained by PC1 (5%). Instead, PCs 3–10 explain 31% of the total variance. Seedlessness is a valued trait in commercially grown table grapes.^46^ A single grape cultivar ‘Sultanina’ is a primary source of seedlessness in table grapes and is a parent of many commercial seedless table grape varieties.^47,48^ Consistent with these observations, previous work on the accessions studied here found that Sultanina has 28 first-degree relationships (that is, sibling or parent–offspring) with other accessions in our dataset.^26^ The repeated use of ‘Sultanina’ in the breeding of seedless accessions, and the resulting high degree of relatedness among all seedless accessions, is a likely contributor to the correlation between seedlessness and population structure observed here.

**Figure 5:**
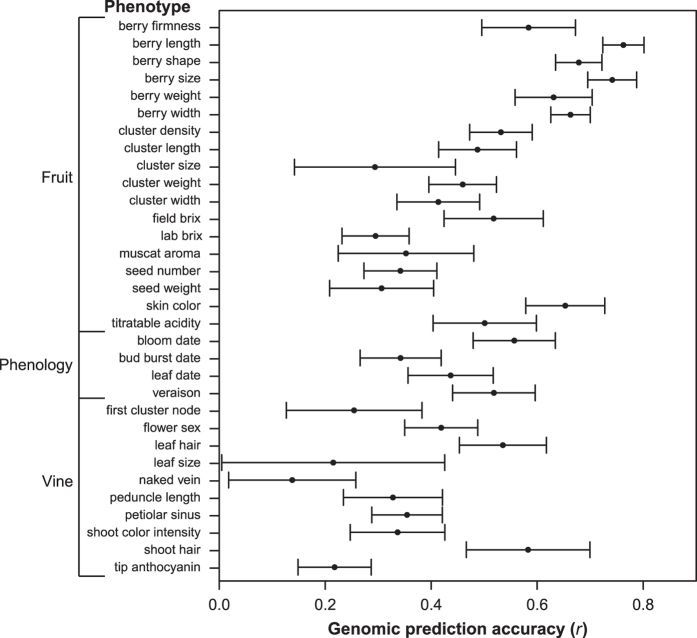
Genomic prediction accuracy for each phenotype. *r-* values represent the correlation between observed and predicted phenotype scores (+/− standard deviation) using a fivefold cross-validation procedure and rrBLUP model repeated three times.

An extension of using PCs to explain phenotypic variance is to perform genomic prediction, which uses all markers to predict phenotypes. Especially for complex traits controlled by numerous small effect loci, genomic prediction is emerging as a powerful tool in genomics-assisted breeding.^49^ Using fivefold cross-validation, we calculated prediction accuracies for all phenotypes (Figure 5 and Supplementary Table S4). Prediction accuracies (*r*) range from 0.10 for leaf size to 0.76 for berry length. We detected the highest prediction accuracies for phenotypes describing berry traits including berry length (0.76), size (0.74), shape (0.68), width (0.66), skin color (0.65), weight (0.63) and firmness (0.58). These prediction accuracies are slightly higher than those previously reported in apple and rice, which had a maximum value of 0.55 for harvest season and 0.63 for flowering time, respectively.^24,50^ Complex quantitative traits such as those describing berry shape and size are better targets for improvement through genomic prediction than from single marker MAS. A genomics-assisted breeding scheme in which both MAS and GS are incorporated has been proposed in apple and may be a viable option in order to select for both monogenic and polygenic traits in grape.^51^

Finally, similar to previous work in apple by Migicovsky *et al.*,^24^ genomic prediction accuracy was also highly correlated with the proportion of phenotypic variance explained by genetic PCs 1–10 (*r*=0.87, *P*=4.23×10^−11^; Supplementary Figure S4). Given that both methods capture genetic relatedness among accessions, a significant relationship between these two methods is expected.

### GWAS and selection scans

Principal component analysis (Figure 3b) revealed significant population structure defined by geography and use, but strong signals of genetic structure also exist at the level of pedigree relatedness. Previous work determined that 75% of the accessions evaluated here are related to at least one other accession by a first-degree relationship, and over half of the accessions are inter-related and form a single, complex pedigree network.^26^ Both the strong population-level and pedigree-level signals of genetic structure in our sample present challenges in genetic mapping as these are significant confounding factors when performing GWAS. Moreover, the rapid LD decay previously described for this and other diverse populations of *V. vinifera* suggests that millions of SNPs are required for well-powered GWAS in grapes.^26,52^ Despite our relatively low marker density and the challenges presented by strong genetic structure, we performed GWAS for all 33 phenotypes. For most traits, we found no convincing GWAS signals (Supplementary Table S5 and Supplementary Figure S5). However, we reasoned that we may find SNPs associated with key traits that experienced strong selection during domestication and breeding because selection results in extended LD surrounding the targeted loci, thereby requiring a lower SNP density than that required to map-unselected traits. We hypothesized that, by combining association mapping (GWAS) with selective sweep mapping (xpEHH), we may identify loci associated with traits targeted during grape domestication and breeding.

**Figure 6:**
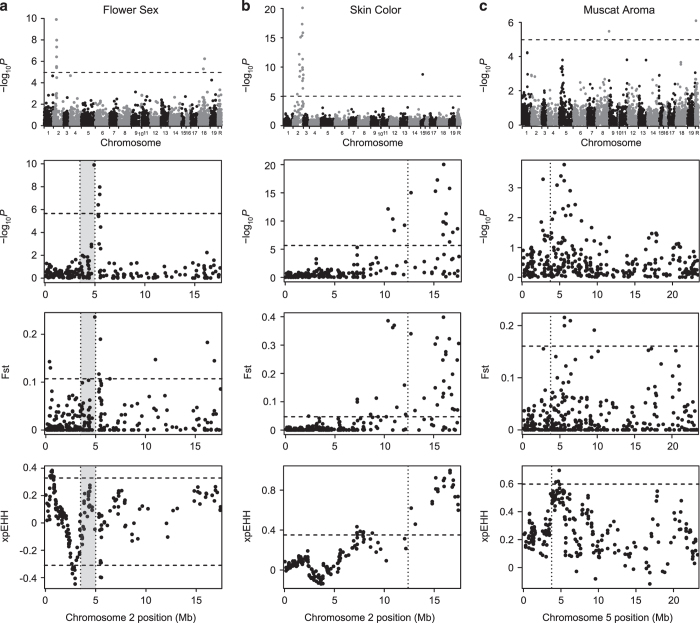
GWAS and selection scan results for (**a**) flower sex, (**b**) skin color and (**c**) Muscat aroma. Full Manhattan plot of GWAS results and Manhattan plot of GWAS results on chromosome with significant result only. *P*-values are log-transformed. The horizontal dotted line indicates a Bonferroni-corrected *P*-value threshold for significance. Chromosome R indicates SNPs found on contigs that remain unanchored to the reference genome. Fst and xpEHH selection scan profiles for corresponding GWAS results on the chromosome of interest. The horizontal dotted lines indicate the top and bottom 1% values for each test across the entire genome. The known loci for flower sex (**a**), skin color (**b**) and muscat aroma (**c**) in grapes are indicated by a vertical dotted line.

A key transition in grapevine domestication was the switch from dioecy to hermaphroditism: all wild *Vitis* species, including the ancestor of *V. vinifera*, are dioecious, and nearly all *V. vinifera* are hermaphroditic. Hermaphroditism was likely the first, and arguably the most important, transition from wild vines to cultivated grapes: it enables self-pollination and subsequent clonal propagation of elite cultivars without the need for pollinators.^53^ Dioecy is found at low frequency in our sample: only 50 of the 550 accessions with flower sex data were labeled as dioecious. Despite this low frequency, we identified SNPs significantly associated with flower sex on chromosome 2 (Figure 6a). The most significantly associated SNP (chr2:4916490) overlaps with the 1.5 Mb region repeatedly identified via linkage mapping.^17,54,55^ This SNP is also found within the fine-mapped 143 kb region (4.91–5.05 Mb) believed to harbor the causal flower sex locus.^56^ We therefore demonstrate that, even with only 50 accessions (9% of the sample) carrying the ancestral dioecy phenotype, we successfully map the flower sex locus at relatively high resolution using GWAS relative to traditional linkage-mapping approaches.

A genome-wide Fst scan comparing dioecious to hermaphroditic accessions also revealed that the SNP most strongly associated with flower sex had the highest Fst value genome-wide, consistent with the effect of selection for hermaphroditism at this locus (Figure 6a). If grape domestication resulted in a rapid increase in the frequency of the hermaphroditism allele, one would expect extended haplotype homozygosity, and thus extremely high xpEHH values, in and around the flower sex locus. While none of the xpEHH values at the flower sex locus fall within the top 1% most extreme values genome-wide, we do observe a suggestive peak with xpEHH values within the top 2.6% of genome-wide xpEHH values (Figure 6a). xpEHH values in the bottom 1% of the genome-wide distribution are found directly adjacent to the flower sex locus identified here. We have no explanation for why a potential signature of selection could exist for dioecy in such close proximity to the flower sex locus. There are SNPs with extreme xpEHH and Fst values, indicating potential selection for hermaphroditism, at the distal end of chromosome 1 ranging from positions 366 to 467 kb (Figure 6a). This genomic region overlaps with the region previously associated with flower sex in a bi-parental mapping population using the same Vitis9KSNP microarray employed for this study.^57^ However, this region is several Mb from the locus highlighted in Figure 6a that has been repeatedly associated with flower sex. We hypothesize that this distal signal of selection is due to inaccurate localization of the array’s SNPs in the reference genome since, when this trait is mapped using genotyping-by-sequencing in the same bi-parental population, the flower sex colocalizes with the known flower sex locus according to the reference genome.^58^ It is unclear why such mismapping occurs with the Vitis9KSNP array data, but unexpected hybridization of non-targeted paralogous regions may possibly contribute to these observations.

Skin color in grapes is largely controlled by a single locus on chromosome 2, where a retrotransposon insertion in the *MYBA1* gene results in a loss in pigmentation by disrupting anthocyanin biosynthesis.^15,59^ Although rare, white-skinned grapes have been observed without this associated retrotransposon insertion, suggesting that this phenotype has arisen via mutation elsewhere in the genome.^60^ We find SNPs significantly associated with skin color within a diffuse peak on chromosome 2 between 10 and 17 Mb (Figure 6b). Although the genomic region containing significant GWAS hits for color overlaps the *VvmybA1* gene, the most significantly associated SNP found here is 3.6 Mb from the known causal mutation. Our inability to map the known color locus with precision is consistent with results from rice^61^ and Arabidopsis^62^ where markers with the strongest association signals were not found directly at known causative loci. Moreover, this result is unsurprising given the relatively low marker density of the SNP array employed here.

While the diffuse association signal for grape color spanning nearly 7 Mb indicates that we have poor mapping resolution for this phenotype, it also suggests the presence of long-range LD potentially caused by selection. Dark skin is the ancestral state in the genus *Vitis,* while white skin color likely arose after the domestication of *V. vinifera* and was subsequently targeted during the breeding of both wine and table grapes.^1^ We observe extreme Fst values and a significant reduction of haplotype diversity in light-skinned grapes that overlap with our observed association signals for skin color (Figure 6b). These observations confirm that there was strong selection for lighter berry pigmentation during grapevine breeding that had a significant impact on patterns of nucleotide diversity at the grape skin color locus.^1^

In addition to flower sex and color, muscat aroma is a phenotype that was likely targeted by breeders and is inherited in a largely Mendelian manner. Grapes with the muscat aroma are characterized by high concentrations of monoterpenoids whose expression is largely controlled by a nonsynonymous mutation in a transcription factor, *DXS*, on chromosome 5.^19,63–65^ We scored muscat aroma as a binary trait by simply assigning the 29 accessions carrying the word ‘muscat’ in their names to one group, and assigning the remaining 491 accessions to another group. The two SNPs exceeding our GWAS significance threshold did not colocate with the known locus, but were instead located on chromosome 8 and an unanchored contig (Figure 6c). However, we observe a suggestive GWAS peak at the muscat locus that does not exceed the Bonferroni-corrected significance threshold. Reasons for a lack of a significant GWAS signal at the known locus may be due to a lack of SNPs in high LD with the causal SNP, a low frequency of the muscat trait in our population (5%) and/or confounding effects resulting from the high degree of relatedness of the muscat varieties studied here.^26^ While GWAS alone would not have allowed us to unequivocally identify the muscat locus without prior knowledge of its location, the presence of strong signals of positive selection for muscat aroma directly adjacent to the *DXS* gene (Figure 6c) provides orthogonal evidence that leads us to conclude that the GWAS peak on chromosome 5 indeed reflects a meaningful genotype–phenotype association.

**Figure 7:**
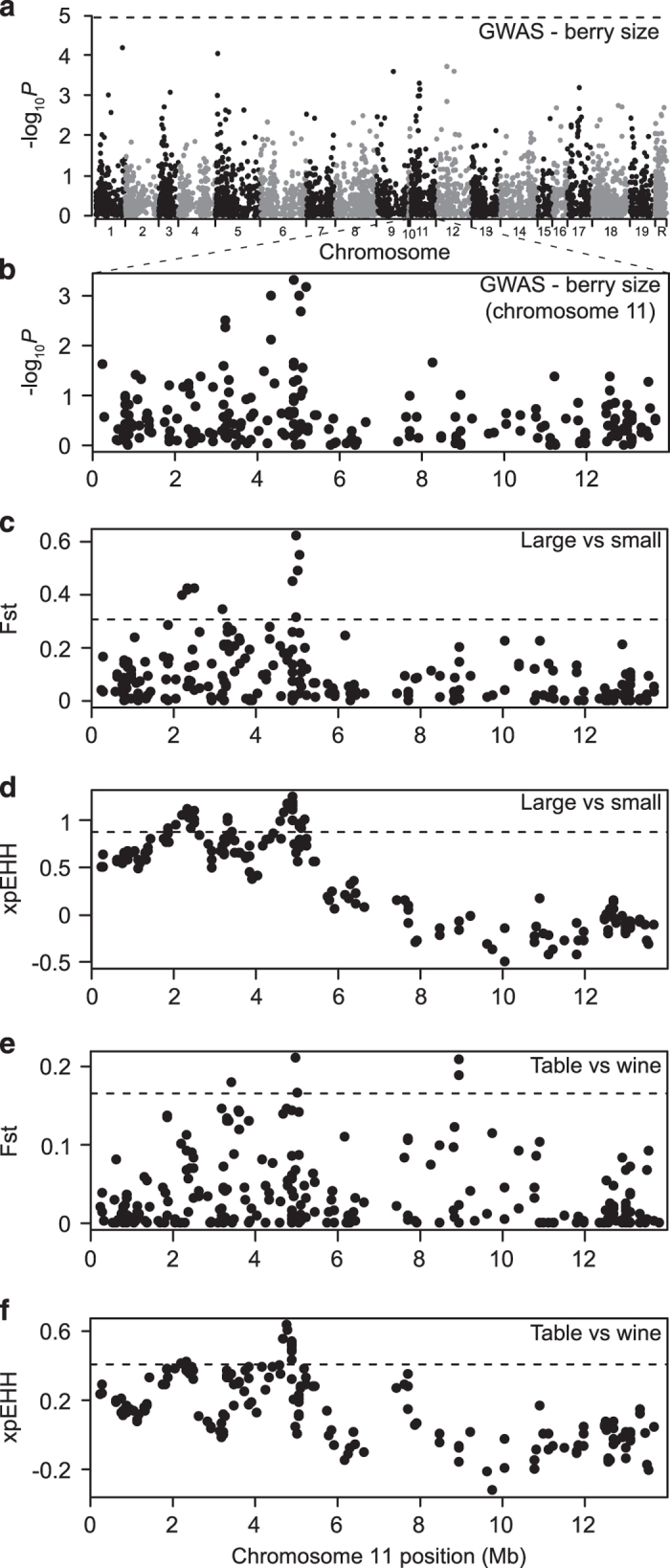
GWAS results for berry size as well as selection scans comparing accessions based on berry size and use. (**a**) Full Manhattan plot of GWAS results for berry size. Chromosome R indicates SNPs found on contigs that remain unanchored to the reference genome. (**b**) Manhattan plot of GWAS results on chromosome 11 only. *P*-values are log-transformed. The horizontal dotted line on GWAS plots indicates a Bonferroni-corrected *P*-value threshold for significance. (**c**) Fst and (**d**) xpEHH selection scan profiles comparing top 10% of largest grapes to 10% smallest grapes. (**e**) Fst and (**f**) xpEHH selection scan profiles comparing table to wine grapes. The horizontal dotted lines for selection scans indicate the top 1% of values for each test across the entire genome.

Our detection of overlapping signals of association and positive selection at the known loci underlying flower sex, color and muscat aroma suggests that combining GWAS and selective sweep mapping can reveal genomic regions underlying traits targeted by grape breeders. Our observation of marked differences in berry size between table and wine grapes suggests that large berry size was likely also a target of selection during table grape breeding. A GWAS for berry size did not result in any SNPs exceeding our significance threshold (Figure 7a). We reason, however, that some of the observed GWAS peaks may represent true genotype–phenotype associations that fail to reach significance in the same manner as described above for muscat aroma. In this case, it is likely that the strong correlation between berry size and population structure (Figure 3d and Figure 4) is largely responsible for the lack of significant GWAS hits for berry size. Without correcting for the confounding effects of population structure, a naive GWAS for berry size (that is, a Pearson correlation between genotypes and phenotypes) results in 75% of SNPs being significantly associated with berry size after Bonferonni correction (Supplementary Figure S4). Thus, when correcting for this strong genotype–phenotype covariance, mixed-model GWAS may, in fact, overcorrect and result in a lack of power to detect true genotype–phenotype associations.

Given the difficulty of mapping berry size using GWAS, we aimed to identify suggestive peaks that also show evidence of positive selection. Of the peaks identified in Figure 7a, we highlight a region on chromosome 11 where the association signal overlaps with signatures of selection (Figures 7b–d). Within this region, we find a reduction of haplotype diversity in large grapes relative to small grapes. Similarly, table grapes show a signature of selection relative to wine grapes (Figures 7e and f). There are two genes that fall within 10 kb of the most significant SNP at chr11:4887417 for berry size. Both GSVIVT00016927001 and GSVIVT00016928001 have GO terms for copper ion binding, electron transport and electron carrier activity. It is unclear what functional role, if any, these genes may have in berry size, but these two candidates may be worthy of future investigation.

We demonstrated that the primary axis of genetic structure differentiates wine from table grapes (Figures 3b and c), and that berry size is also strongly correlated with the first genetic PC (Figure 3d). It is clear that geographic isolation is at least partially responsible for differences between Eastern table grapes and Western wine grapes. However, the reduction in genetic diversity at this locus on chromosome 11 provides evidence that the size difference between table and wine grapes may not be due to geography alone but may have been driven by selection for larger table grapes in the East. Whether berry size became a breeding target was largely a result of the predominant religion in the geographic area in which the grapes were bred: table grapes were bred to be large in the East where Islam predominates and alcohol was prohibited, while wine grapes retained the ancestral smaller size that is more desirable for winemaking in predominantly Christian regions in the West. Thus, we demonstrate that religious rules concerning alcohol consumption not only shaped genome-wide patterns of genetic variation in grapes, but may have shaped patterns of nucleotide diversity within a genomic region associated with berry size.

## Conclusion

Gene banks are often characterized phenotypically and the data are frequently made publicly available. An analysis of historical phenotype data collected over a 17-year period from the USDA grape germplasm collection revealed novel insights into patterns of grape phenotypic diversity, and enabled high-resolution genetic mapping when paired with genomic data. LD decays rapidly in grapes and the SNP density in the current study is arguably inadequate for well-powered GWAS. However, we demonstrate that a modest number of genetic markers is sufficient to uncover loci targeted during domestication and breeding because of the extended LD at these loci caused by positive selection for hermaphroditism, lighter skin pigmentation and muscat aroma. We extend this reasoning to uncover a novel locus associated with berry size that harbors a signature of selection, and suggest that patterns of nucleotide diversity at this locus have been shaped by table grape breeders selecting for larger berries predominantly in regions where alcohol consumption has been prohibited. The present study reveals how religious rules concerning alcohol consumption have had a marked impact on patterns of phenotypic and genetic diversity in grapes, thus highlighting the powerful role of human culture in shaping the genomes and phenomes of agricultural species.
