# Supplementary material for: Patterns of genomic and phenomic diversity in wine and table grapes
Source: Hortic Res. 2017 Aug 2;4:17035–. doi: 10.1038/hortres.2017.35 (PMC5539807; doi:10.1038/hortres.2017.35)
Supplement: Supplementary Figure S1 [file hortres201735-s1.pdf]

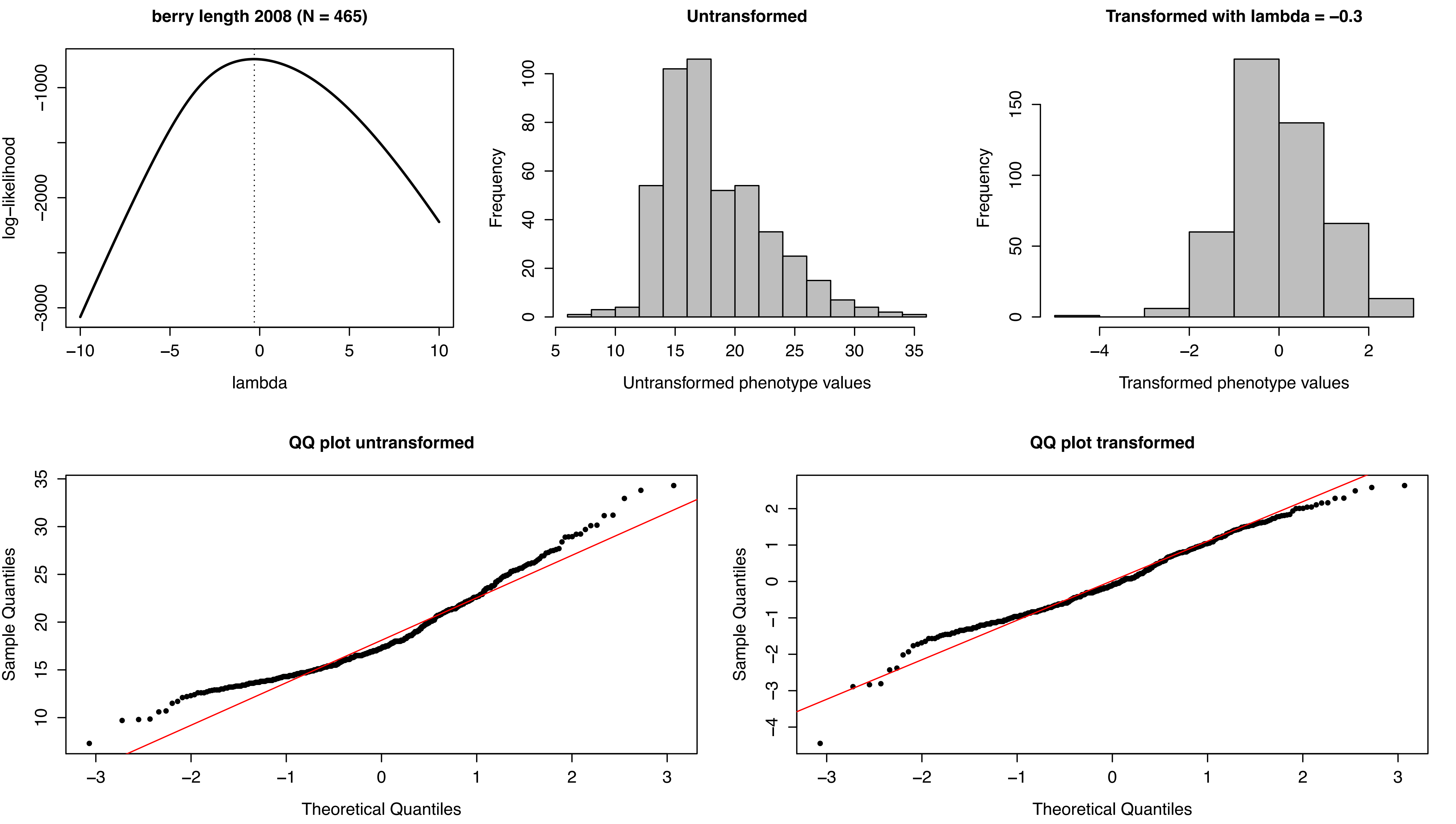
berry length 2008 (N = 465)
log−likelihood
lambda
Untransformed
Frequency
Untransformed phenotype values
Transformed with lambda = −0.3
Frequency
Transformed phenotype values
QQ plot untransformed
Sample Quantiles
Theoretical Quantiles
QQ plot transformed
Sample Quantiles
Theoretical Quantiles

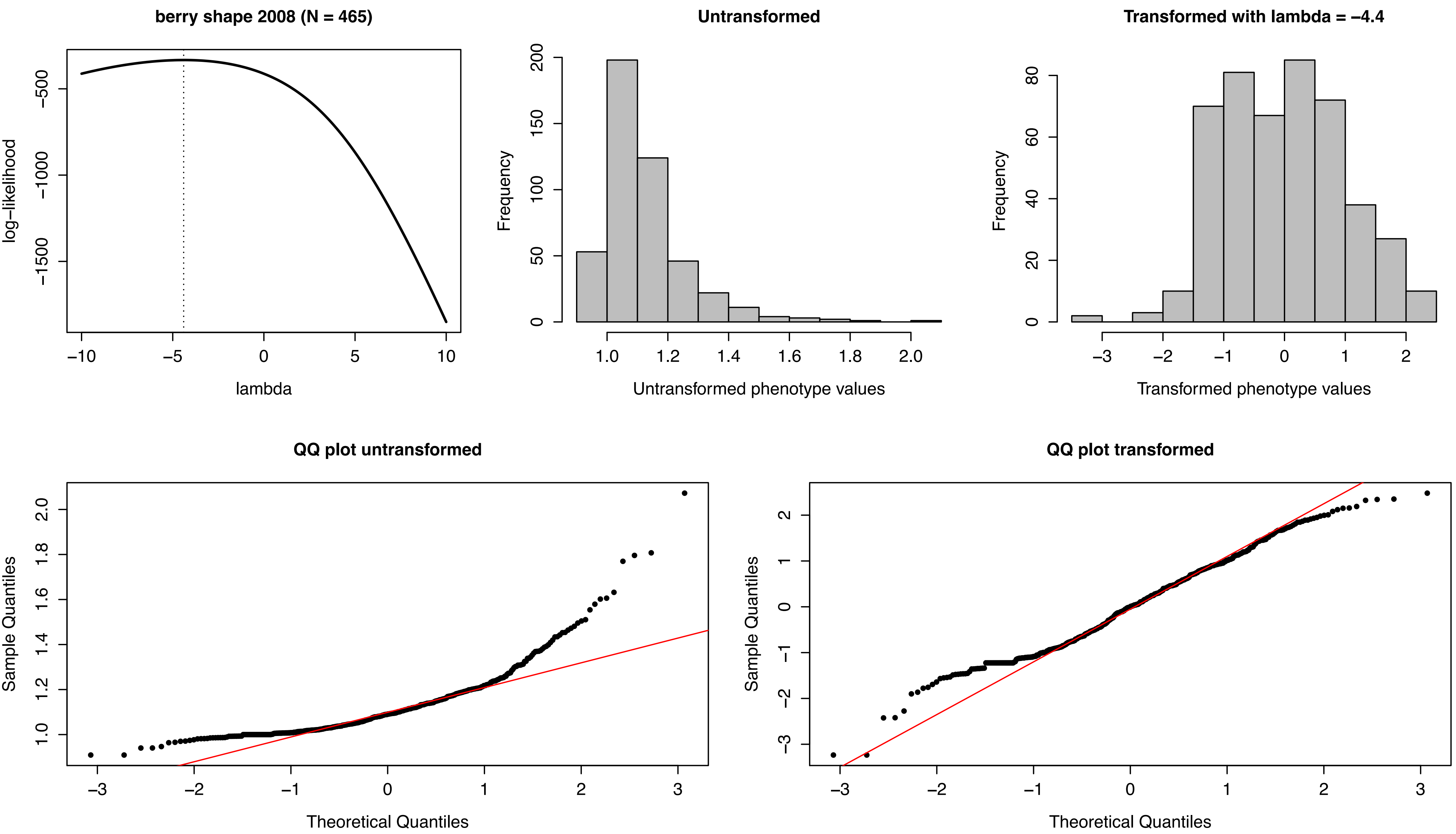
berry shape 2008 (N = 465)
log−likelihood
lambda
Untransformed
Frequency
Untransformed phenotype values
Transformed with lambda = −4.4
Transformed phenotype values
QQ plot untransformed
Sample Quantiles
Theoretical Quantiles
QQ plot transformed

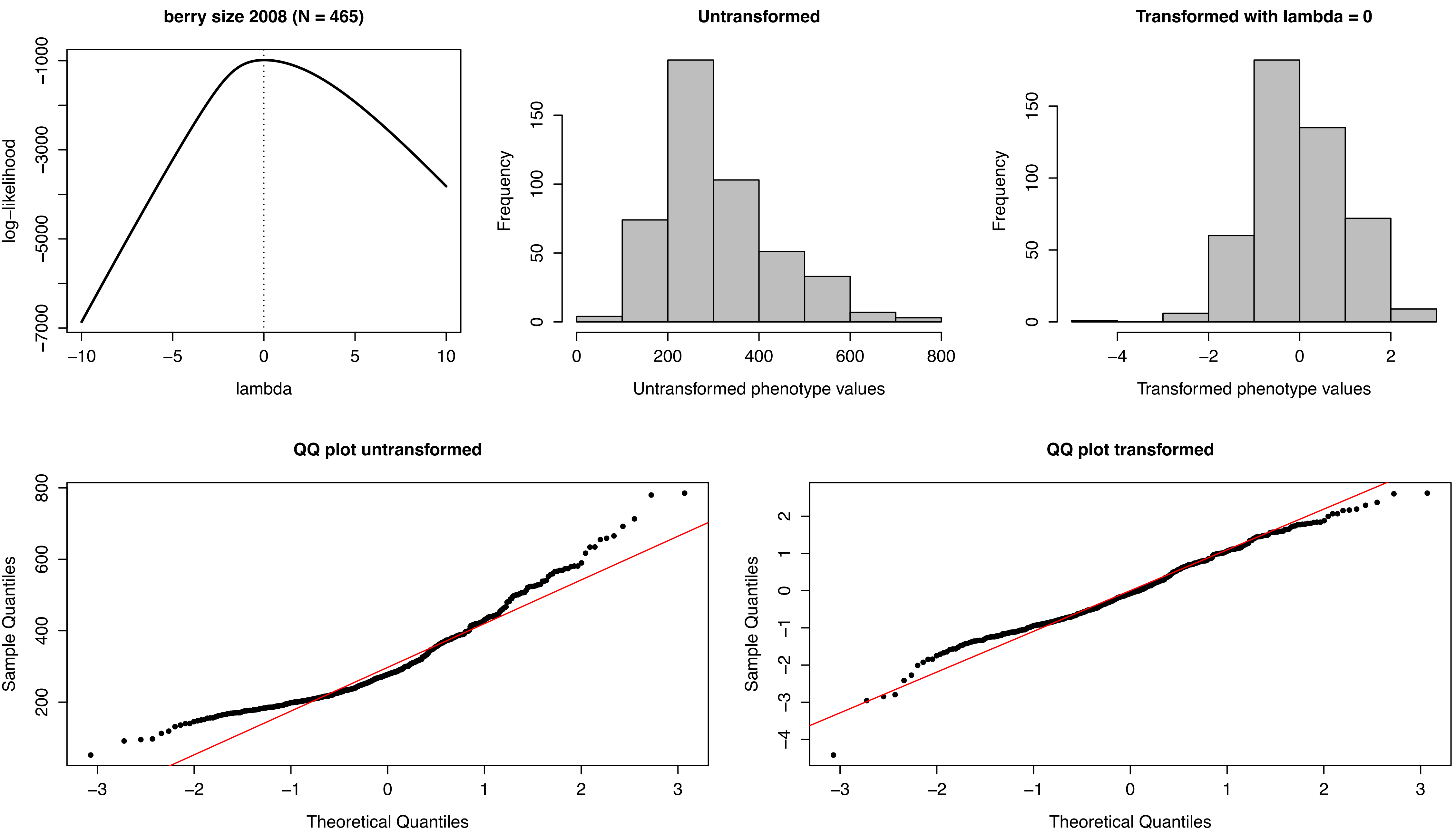

berry size 2008 (N = 465)
log−likelihood
lambda
Untransformed
Frequency
Untransformed phenotype values
Transformed with lambda = 0
Frequency
Transformed phenotype values
QQ plot untransformed
Sample Quantiles
Theoretical Quantiles
QQ plot transformed
Sample Quantiles
Theoretical Quantiles

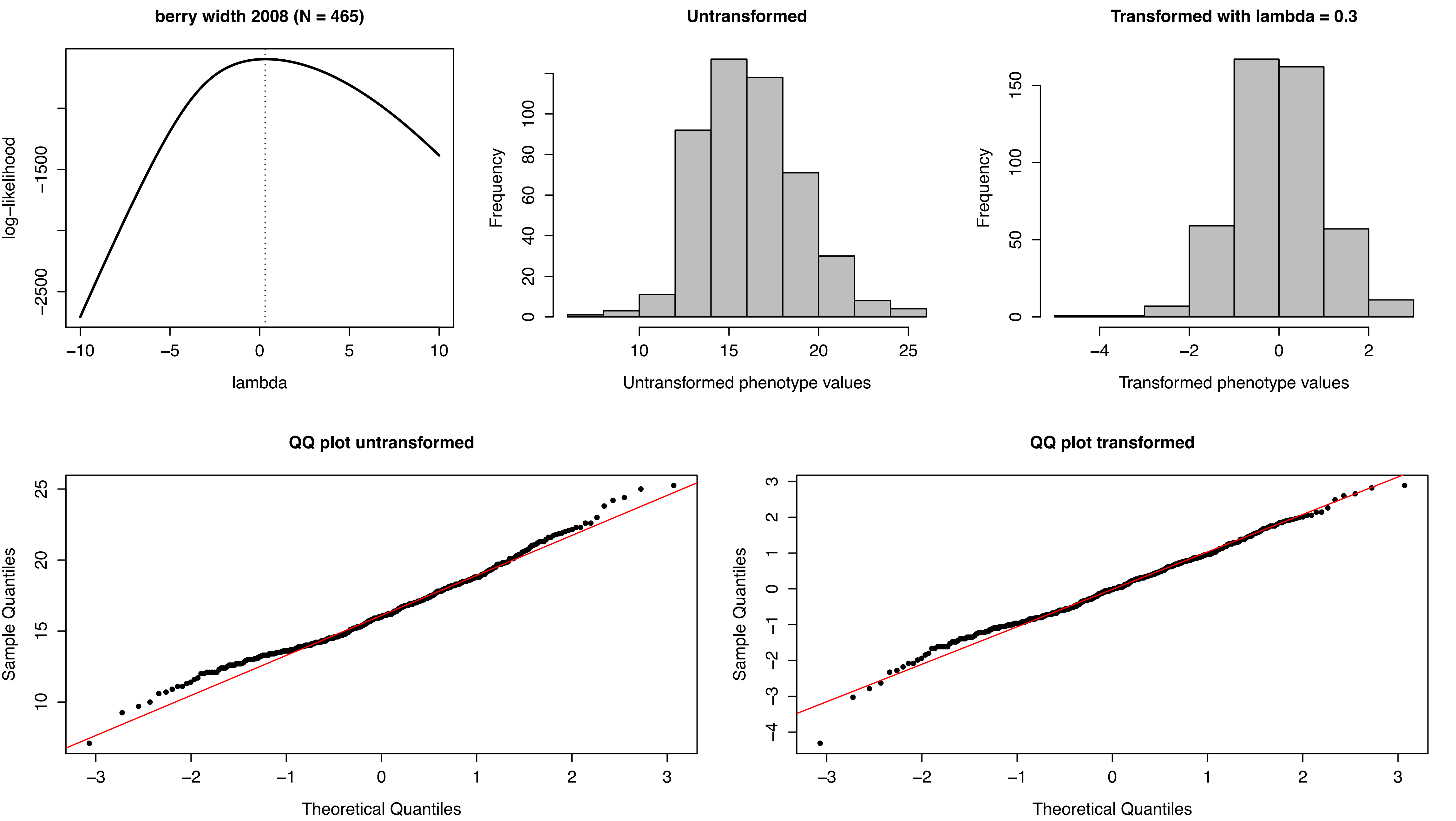

berry width 2008 (N = 465)
log−likelihood
−2500
−1500
−10
−5
0
5
10
lambda
Untransformed
Frequency
0
20
40
60
80
100
10
15
20
25
Untransformed phenotype values
Transformed with lambda = 0.3
Frequency
0
50
100
150
−4
−2
0
2
Transformed phenotype values
QQ plot untransformed
Sample Quantiles
10
15
20
25
−3
−2
−1
0
1
2
3
Theoretical Quantiles
QQ plot transformed
Sample Quantiles
−4
−3
−2
−1
0
1
2
3
−3
−2
−1
0
1
2
3
Theoretical Quantiles

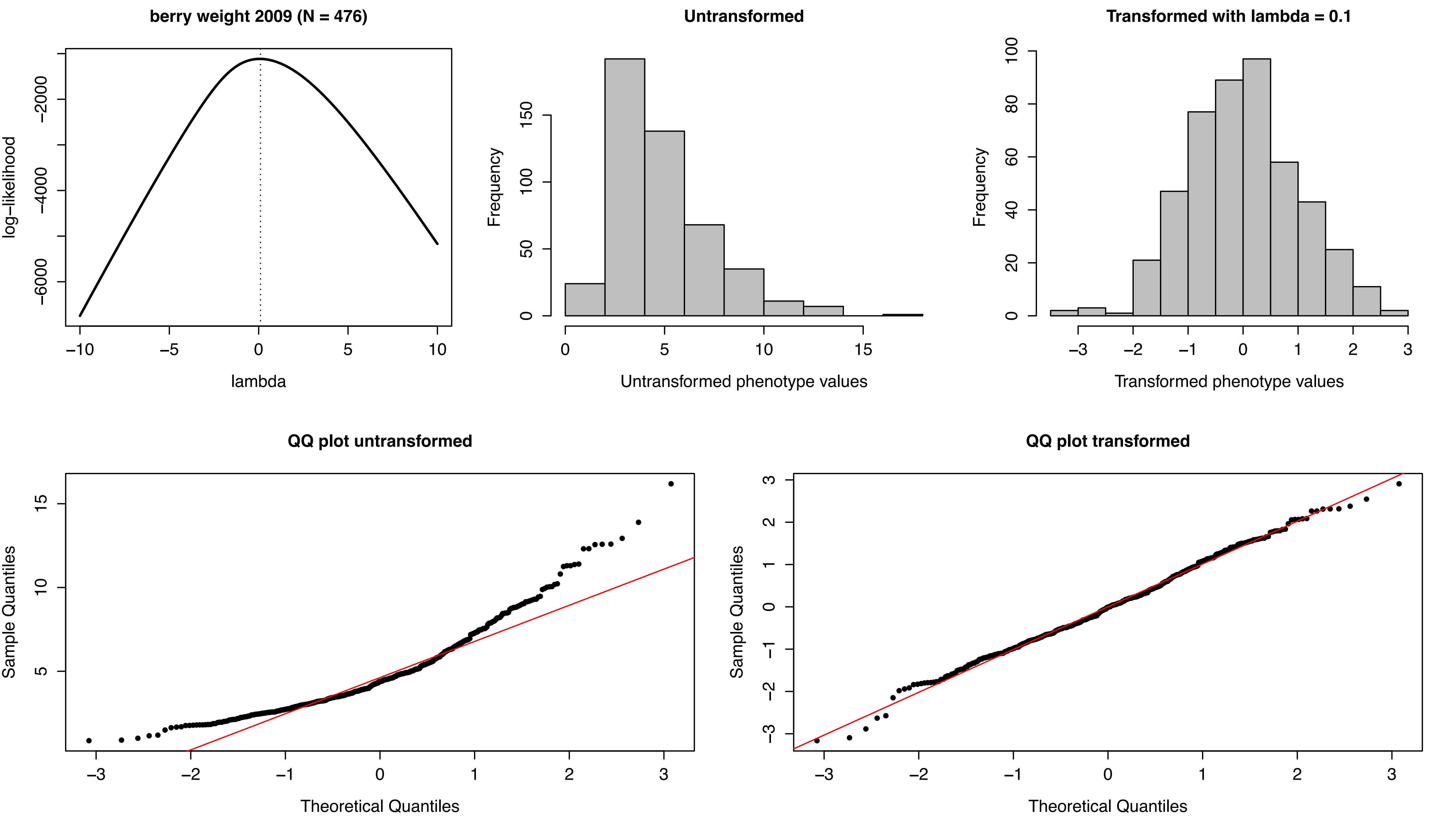
berry weight 2009 (N = 476)
log−likelihood
lambda
Untransformed
Frequency
Untransformed phenotype values
Transformed with lambda = 0.1
Frequency
Transformed phenotype values
QQ plot untransformed
Sample Quantiles
Theoretical Quantiles
QQ plot transformed
Sample Quantiles
Theoretical Quantiles

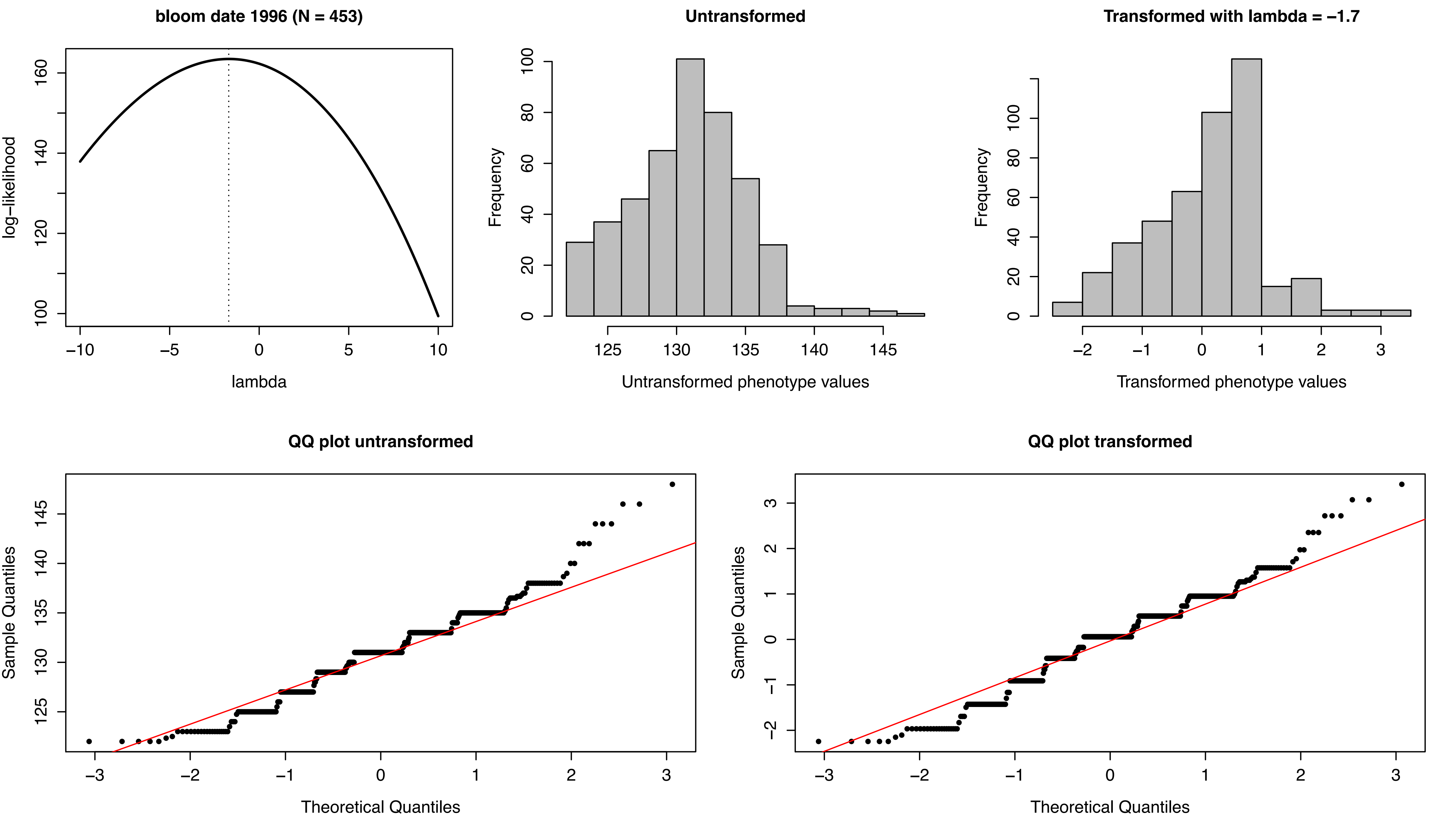
bloom date 1996 (N = 453)
log−likelihood
lambda
Untransformed
Frequency
Untransformed phenotype values
Transformed with lambda = −1.7
Frequency
Transformed phenotype values
QQ plot untransformed
Sample Quantiles
Theoretical Quantiles
QQ plot transformed
Sample Quantiles
Theoretical Quantiles

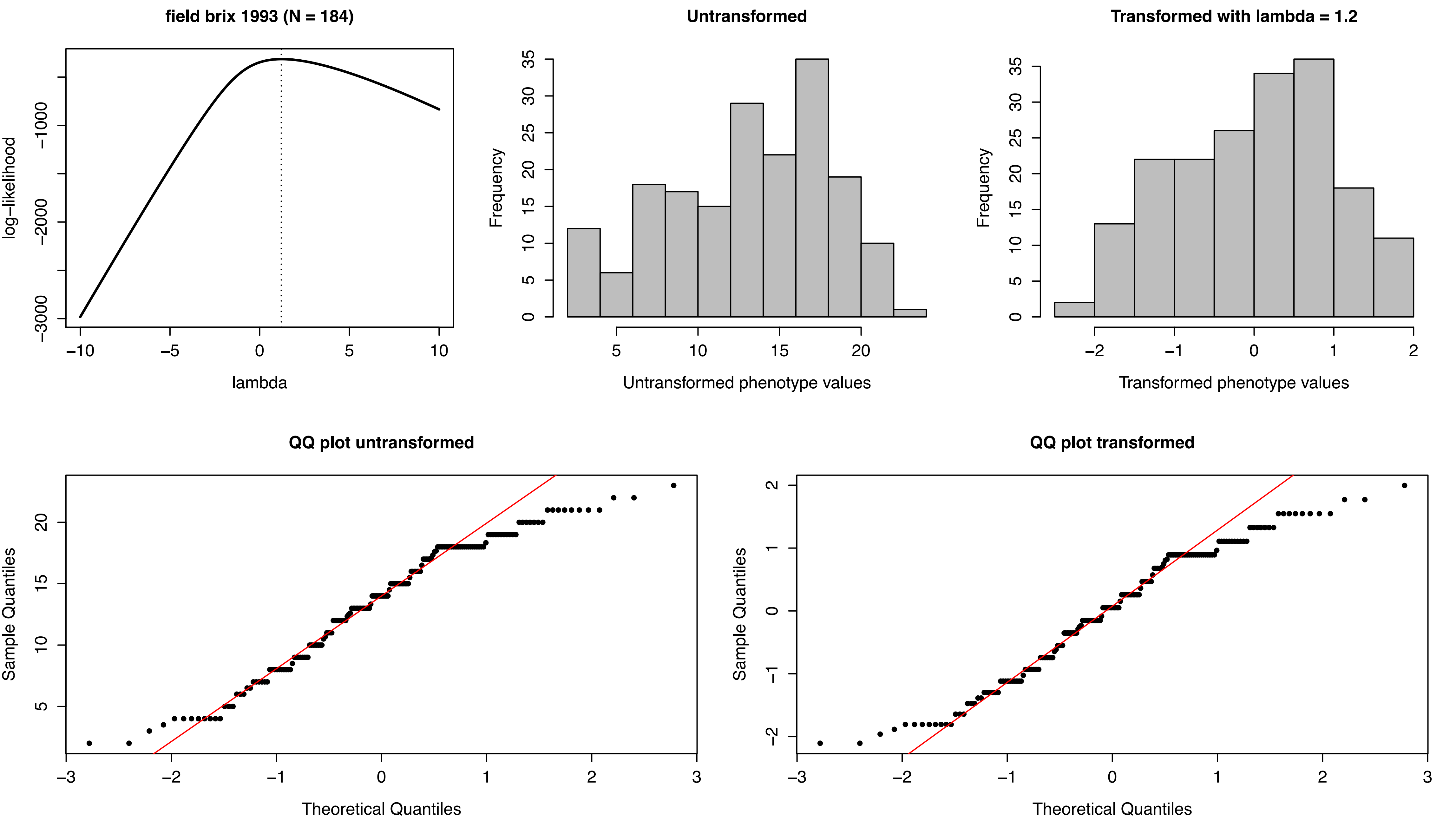

field brix 1993 (N = 184)
log−likelihood
lambda
Untransformed
Frequency
Untransformed phenotype values
Transformed with lambda = 1.2
Frequency
Transformed phenotype values
QQ plot untransformed
Sample Quantiles
Theoretical Quantiles
QQ plot transformed
Sample Quantiles
Theoretical Quantiles

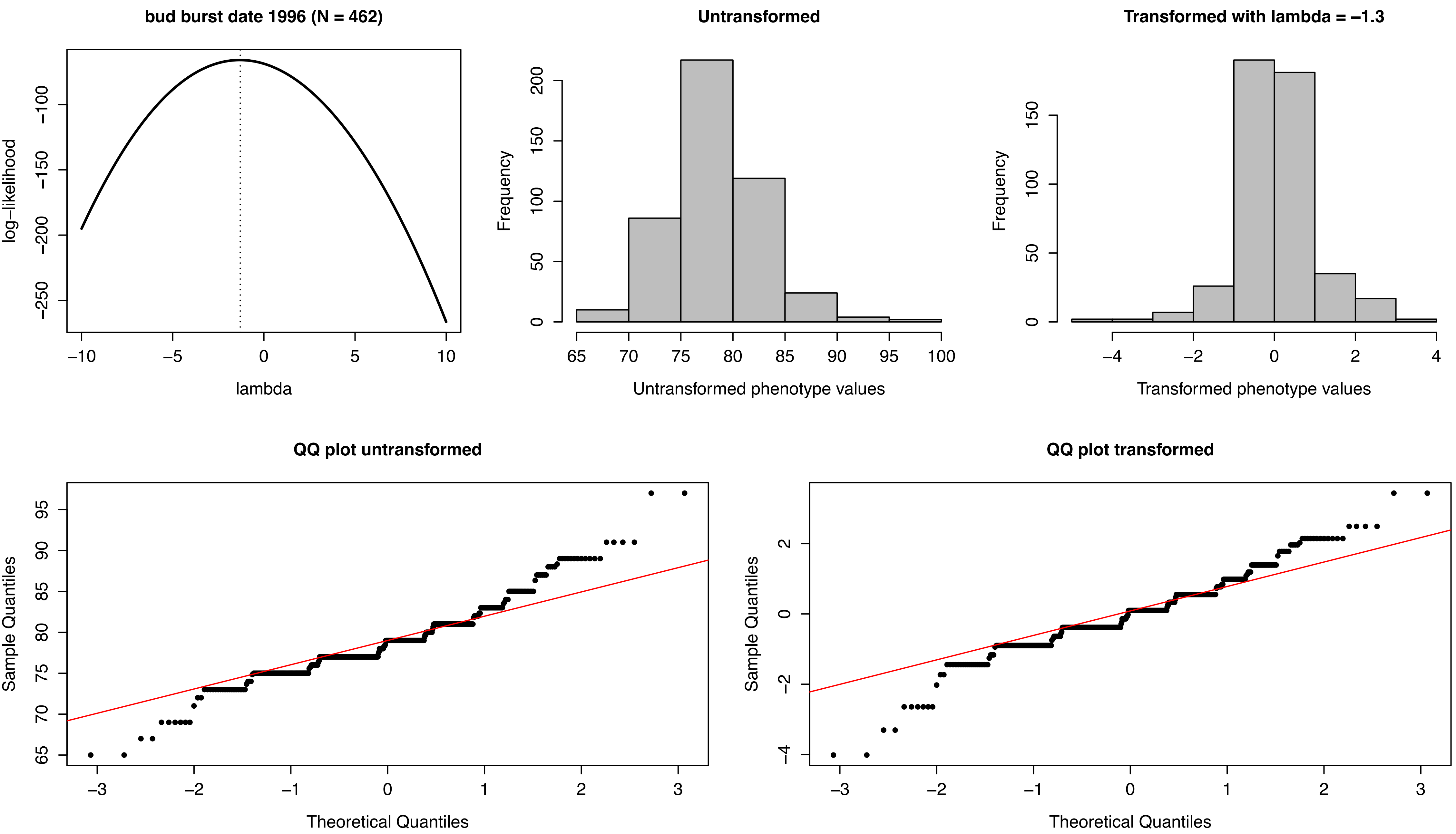
bud burst date 1996 (N = 462)
log−likelihood
lambda
Untransformed
Frequency
Untransformed phenotype values
Transformed with lambda = −1.3
Frequency
Transformed phenotype values
QQ plot untransformed
Sample Quantiles
Theoretical Quantiles
QQ plot transformed
Sample Quantiles
Theoretical Quantiles

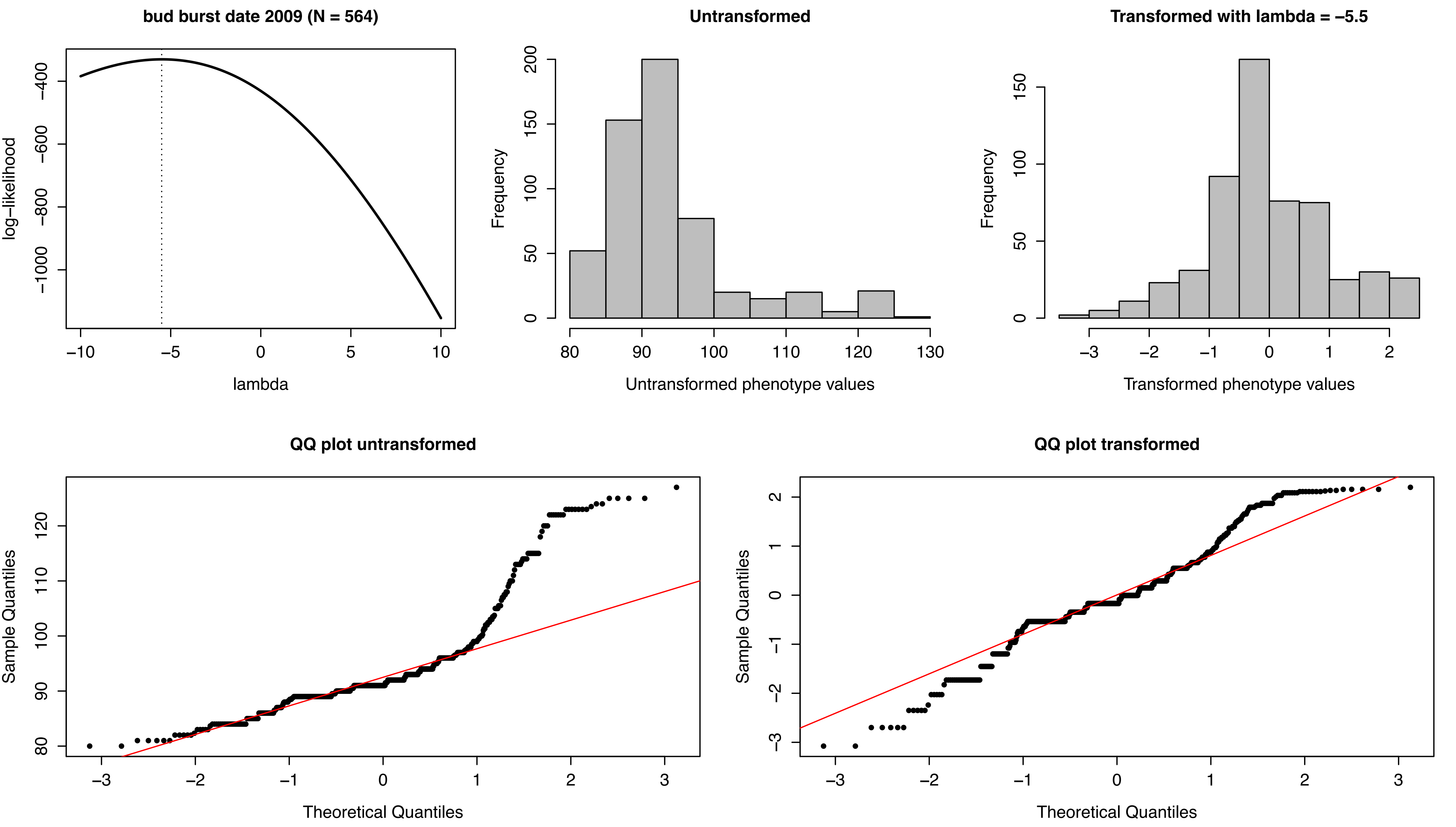

bud burst date 2009 (N = 564)
log−likelihood
lambda
Untransformed
Frequency
Untransformed phenotype values
Transformed with lambda = −5.5
Frequency
Transformed phenotype values
QQ plot untransformed
Sample Quantiles
Theoretical Quantiles
QQ plot transformed
Sample Quantiles
Theoretical Quantiles

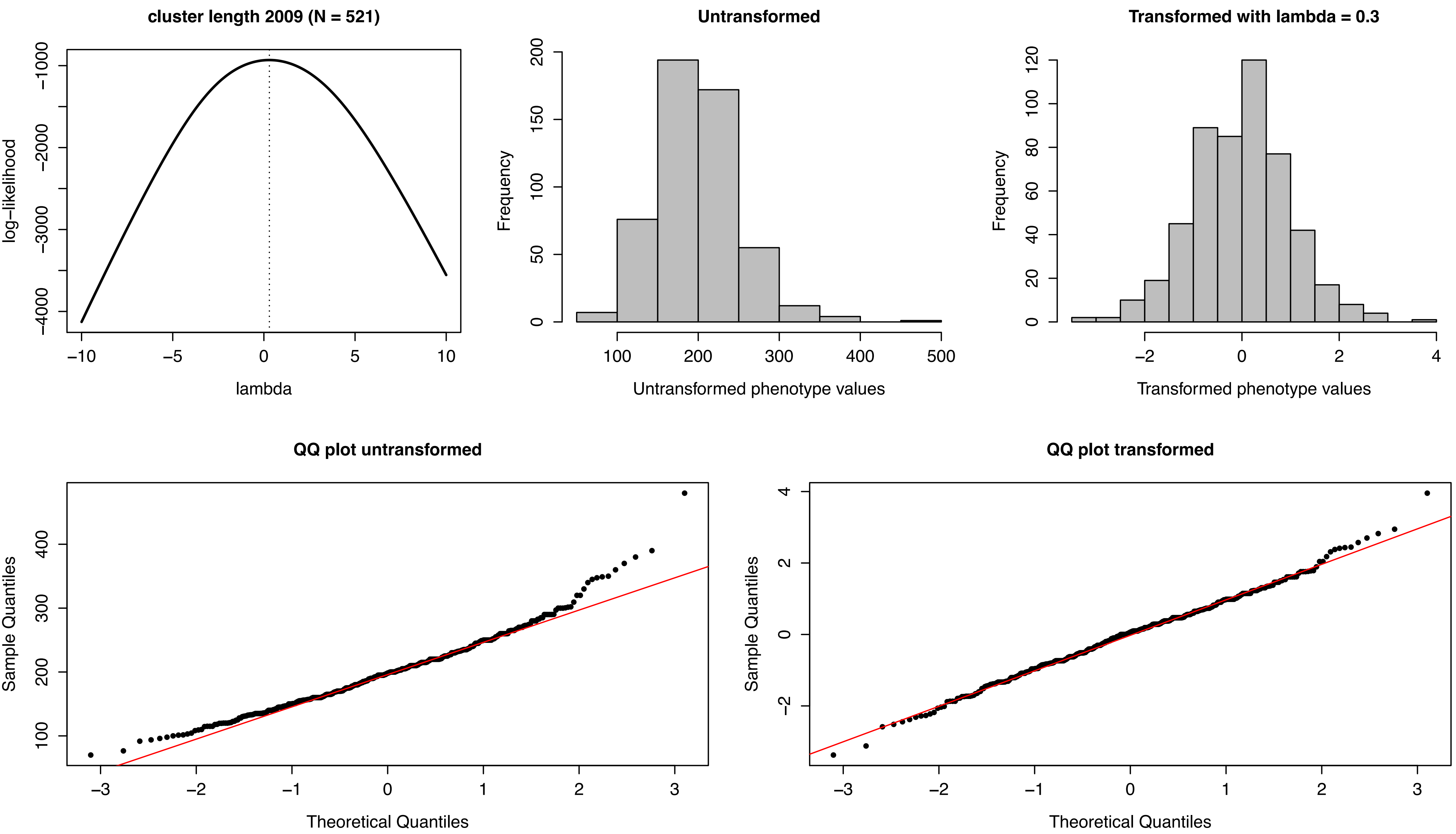
cluster length 2009 (N = 521)
log−likelihood
lambda
Untransformed
Frequency
Untransformed phenotype values
Transformed with lambda = 0.3
Frequency
Transformed phenotype values
QQ plot untransformed
Sample Quantiles
Theoretical Quantiles
QQ plot transformed
Sample Quantiles
Theoretical Quantiles

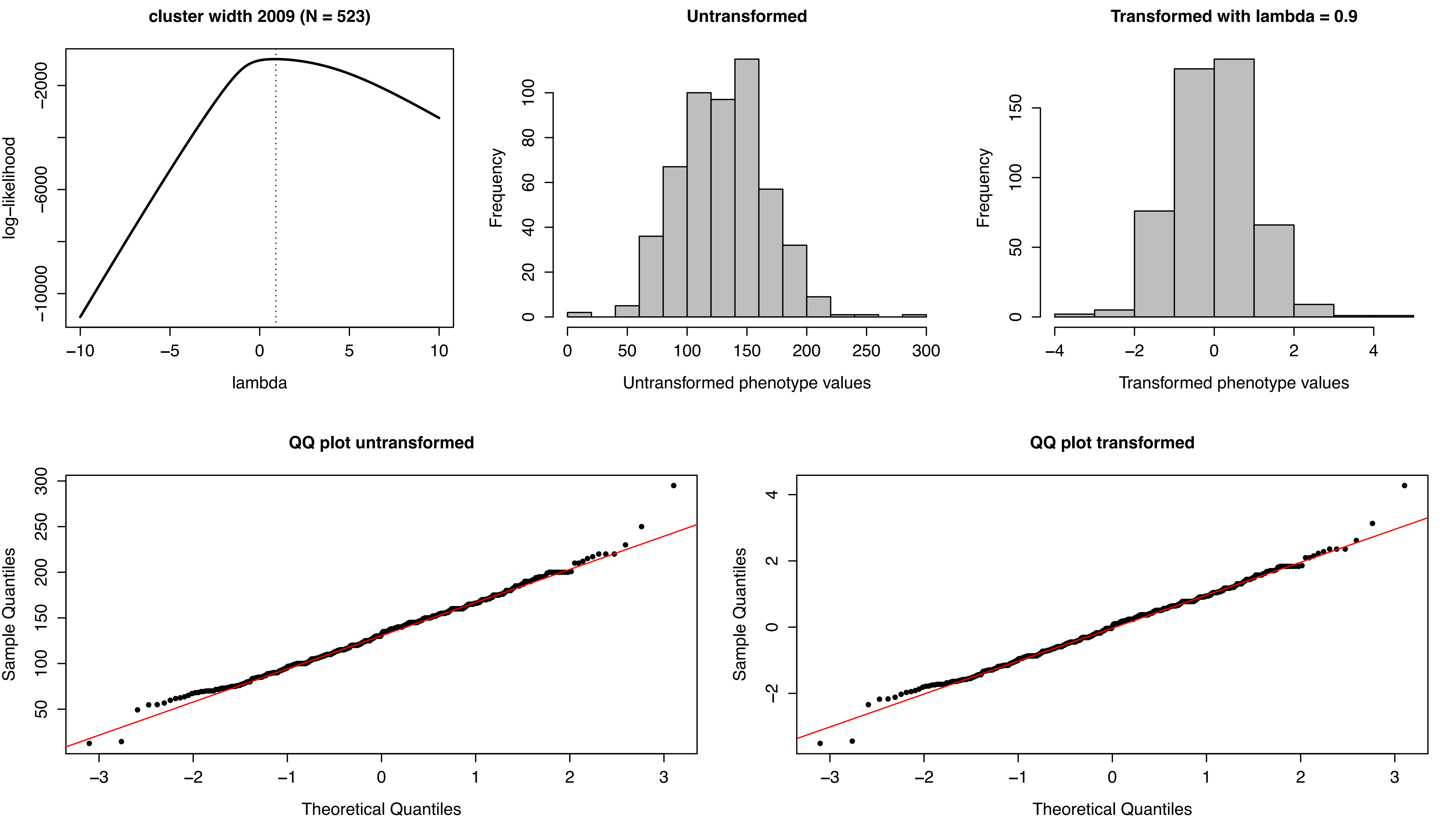

cluster width 2009 (N = 523)
log−likelihood
−2000
−6000
−10000
−10
−5
0
5
10
lambda
Untransformed
Frequency
0
20
40
60
80
100
0
50
100
150
200
250
300
Untransformed phenotype values
Transformed with lambda = 0.9
Frequency
0
50
100
150
−4
−2
0
2
4
Transformed phenotype values
QQ plot untransformed
Sample Quantiles
50
100
150
200
250
300
−3
−2
−1
0
1
2
3
Theoretical Quantiles
QQ plot transformed
Sample Quantiles
−2
0
2
4
−3
−2
−1
0
1
2
3
Theoretical Quantiles

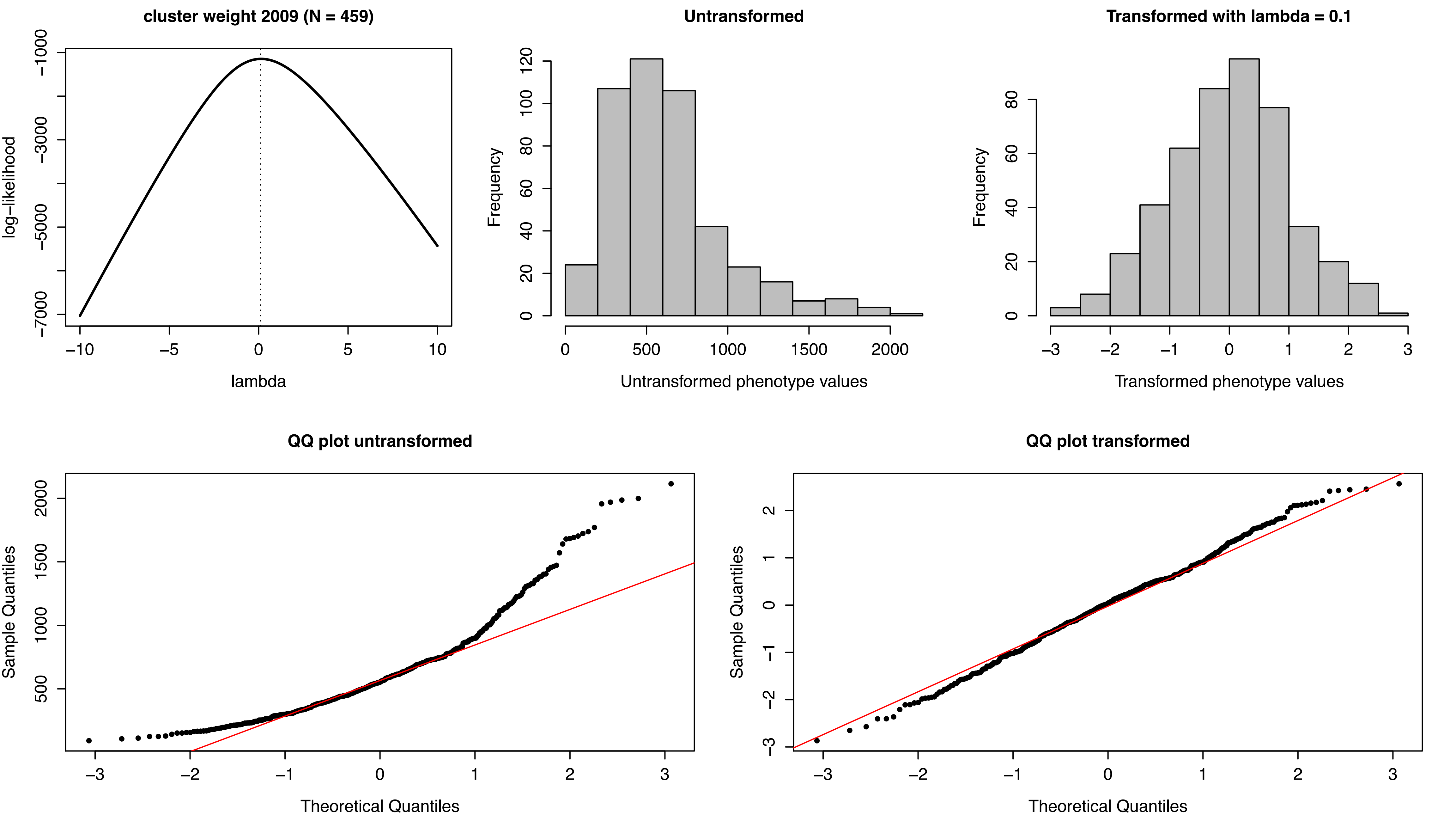

cluster weight 2009 (N = 459)
log−likelihood
−1000
−3000
−5000
−7000
−10
−5
0
5
10
lambda
Untransformed
Frequency
0
20
40
60
80
100
120
0
500
1000
1500
2000
Untransformed phenotype values
Transformed with lambda = 0.1
Frequency
0
20
40
60
80
−3
−2
−1
0
1
2
3
Transformed phenotype values
QQ plot untransformed
Sample Quantiles
500
1000
1500
2000
−3
−2
−1
0
1
2
3
Theoretical Quantiles
QQ plot transformed
Sample Quantiles
−3
−2
−1
0
1
2
−3
−2
−1
0
1
2
3
Theoretical Quantiles

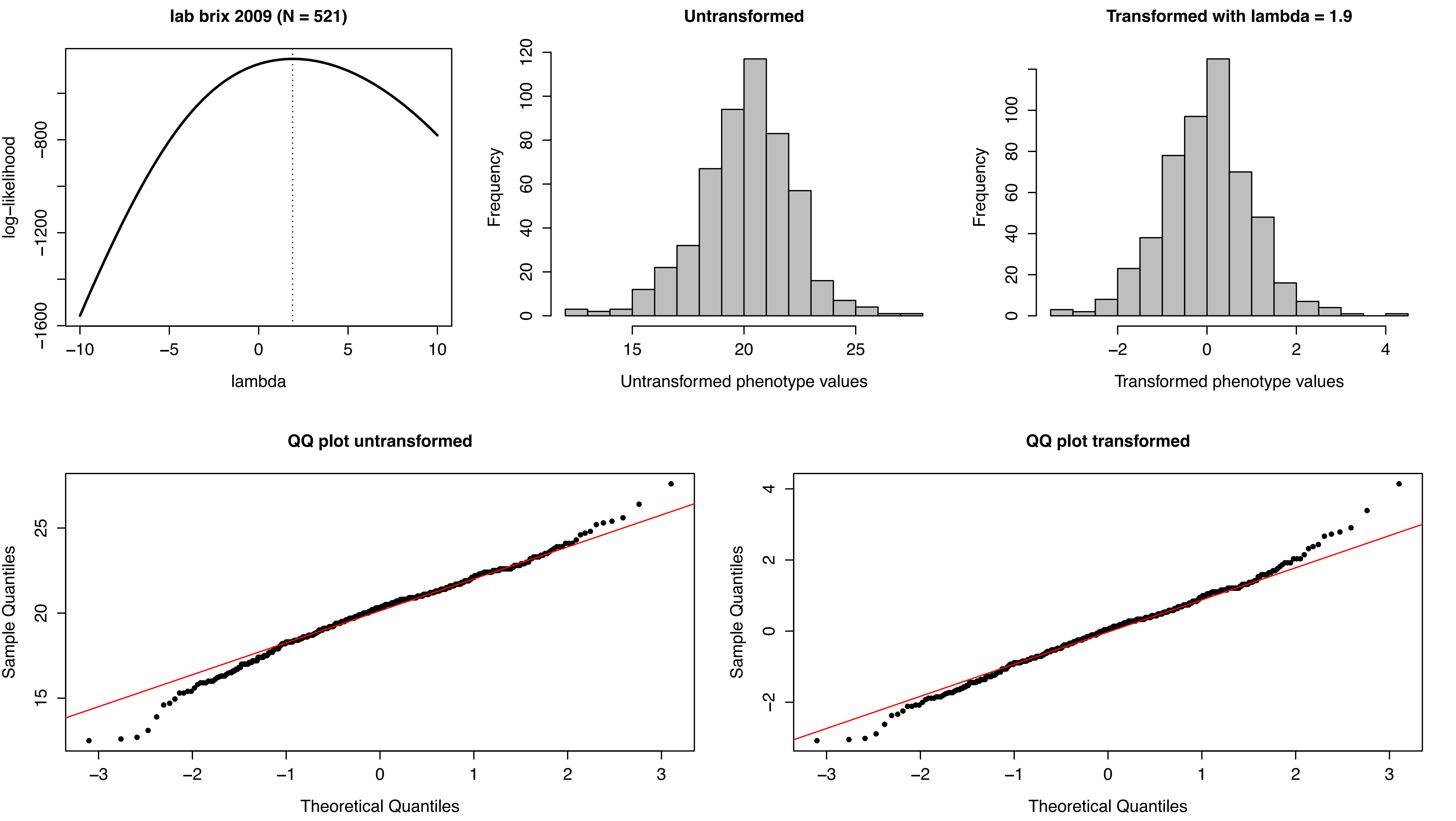

lab brix 2009 (N = 521)
log−likelihood
−1600
−1200
−800
−10
−5
0
5
10
lambda
Untransformed
Frequency
0
20
40
60
80
100
120
15
20
25
Untransformed phenotype values
Transformed with lambda = 1.9
Frequency
0
20
40
60
80
100
−2
0
2
4
Transformed phenotype values
QQ plot untransformed
Sample Quantiles
15
20
25
−3
−2
−1
0
1
2
3
Theoretical Quantiles
QQ plot transformed
Sample Quantiles
−2
0
2
4
−3
−2
−1
0
1
2
3
Theoretical Quantiles

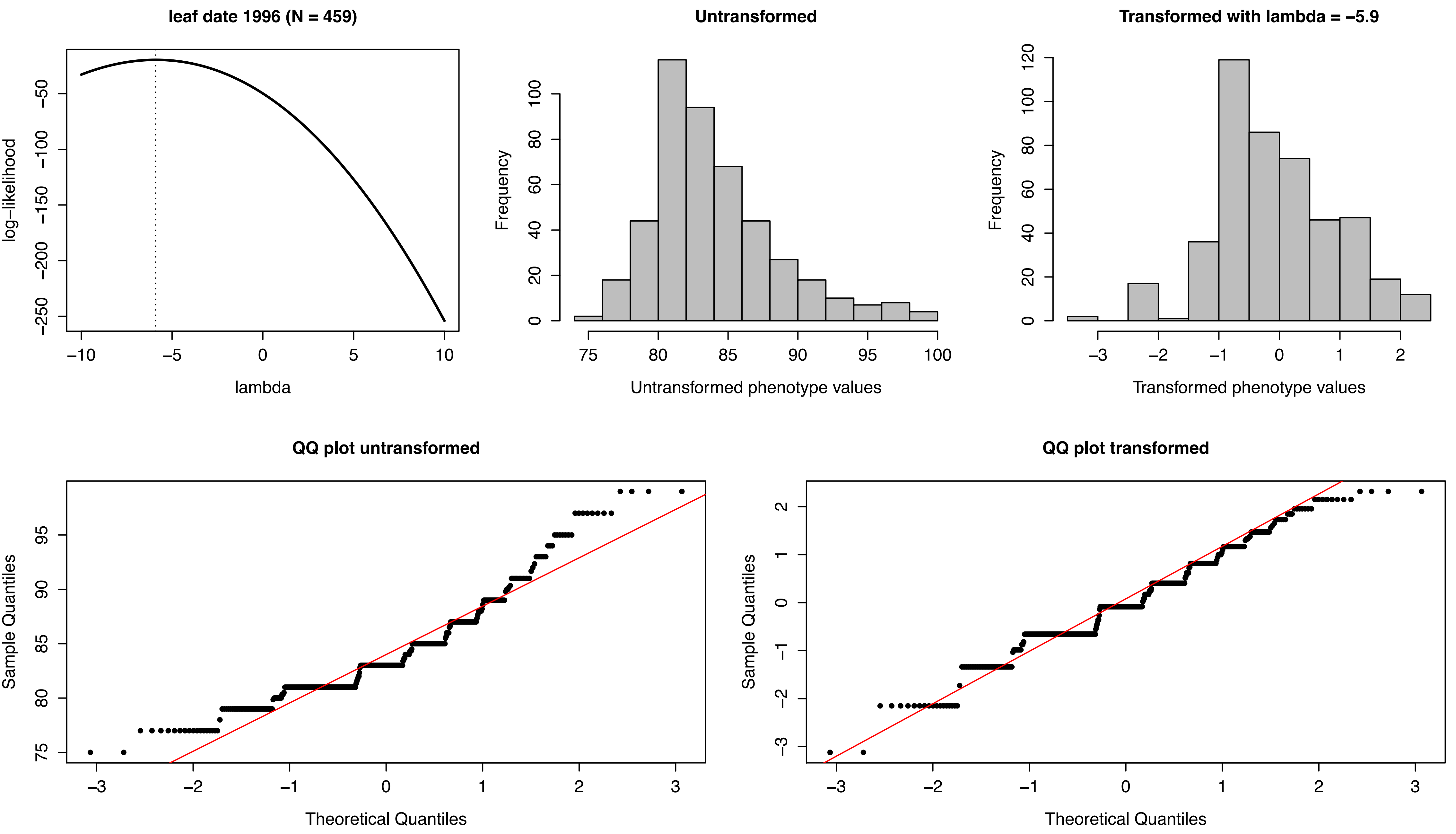

leaf date 1996 (N = 459)
log−likelihood
lambda
Untransformed
Frequency
Untransformed phenotype values
Transformed with lambda = −5.9
Frequency
Transformed phenotype values
QQ plot untransformed
Sample Quantiles
Theoretical Quantiles
QQ plot transformed
Sample Quantiles
Theoretical Quantiles

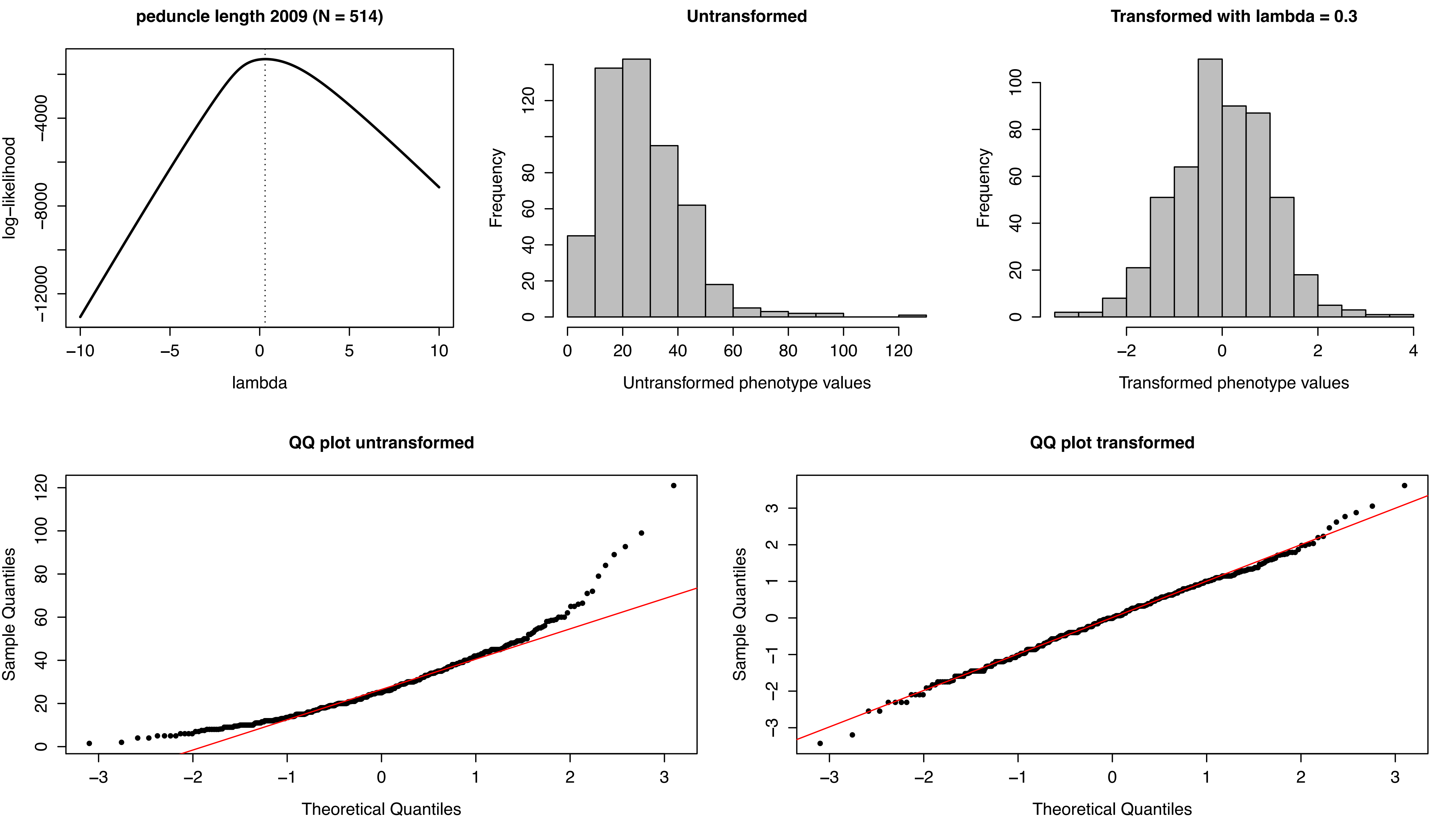

peduncle length 2009 (N = 514)
log−likelihood
lambda
Untransformed
Frequency
Untransformed phenotype values
Transformed with lambda = 0.3
Frequency
Transformed phenotype values
QQ plot untransformed
Sample Quantiles
Theoretical Quantiles
QQ plot transformed
Sample Quantiles
Theoretical Quantiles

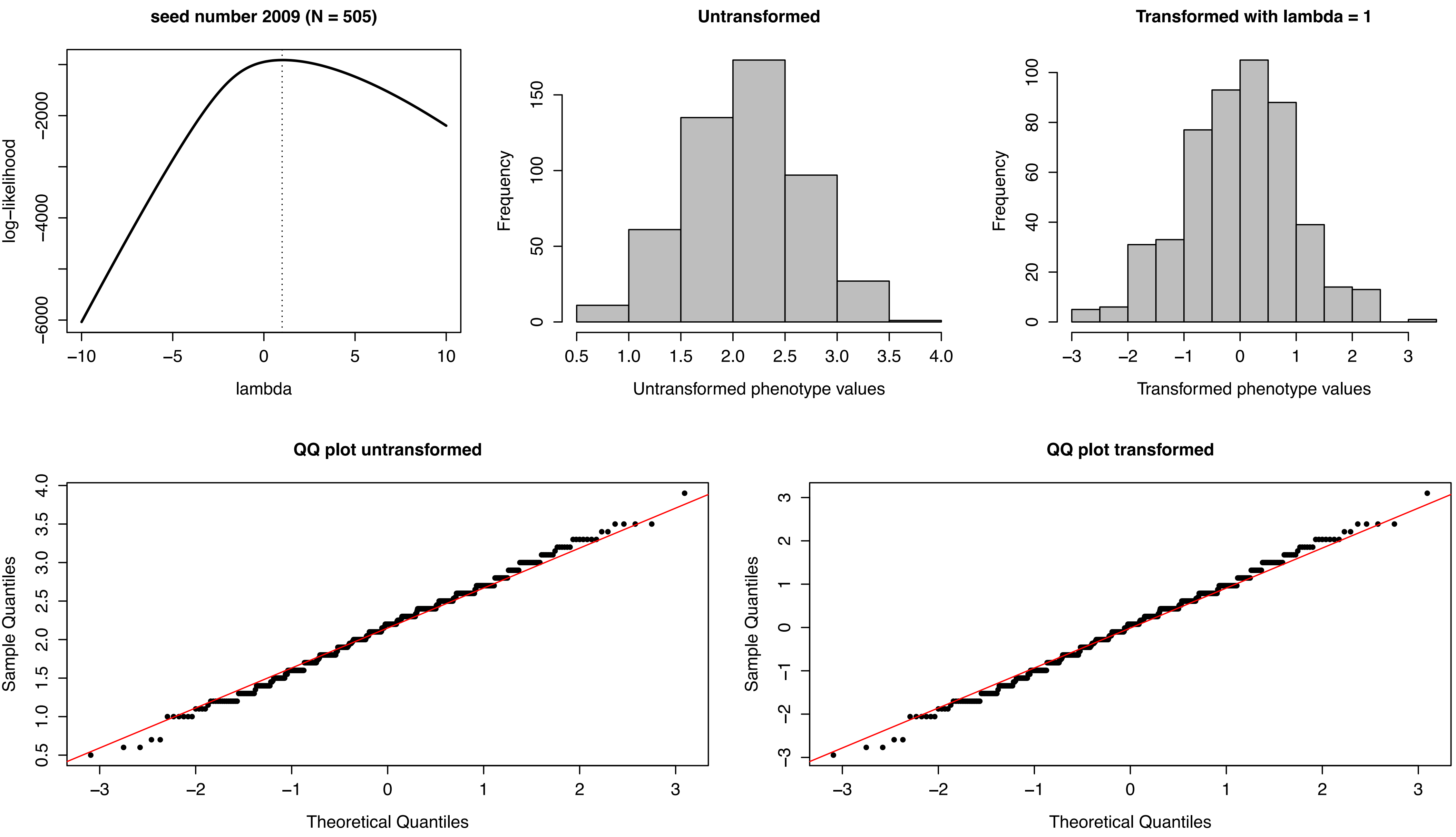
seed number 2009 (N = 505)
log−likelihood
lambda
Untransformed
Frequency
Untransformed phenotype values
Transformed with lambda = 1
Frequency
Transformed phenotype values
QQ plot untransformed
Sample Quantiles
Theoretical Quantiles
QQ plot transformed
Sample Quantiles
Theoretical Quantiles

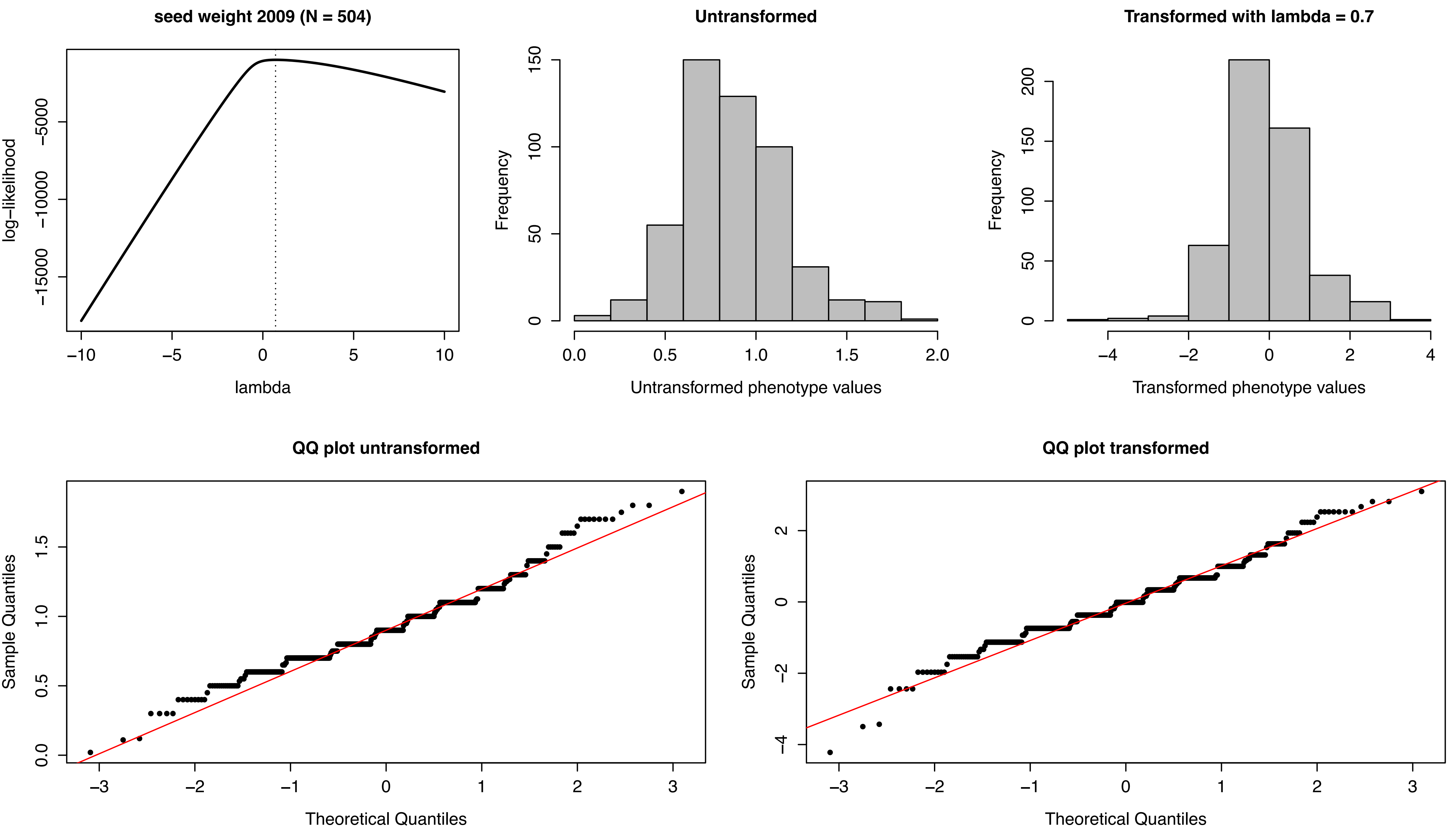
seed weight 2009 (N = 504)
log−likelihood
lambda
Untransformed
Frequency
Untransformed phenotype values
Transformed with lambda = 0.7
Frequency
Transformed phenotype values
QQ plot untransformed
Sample Quantiles
Theoretical Quantiles
QQ plot transformed
Sample Quantiles
Theoretical Quantiles

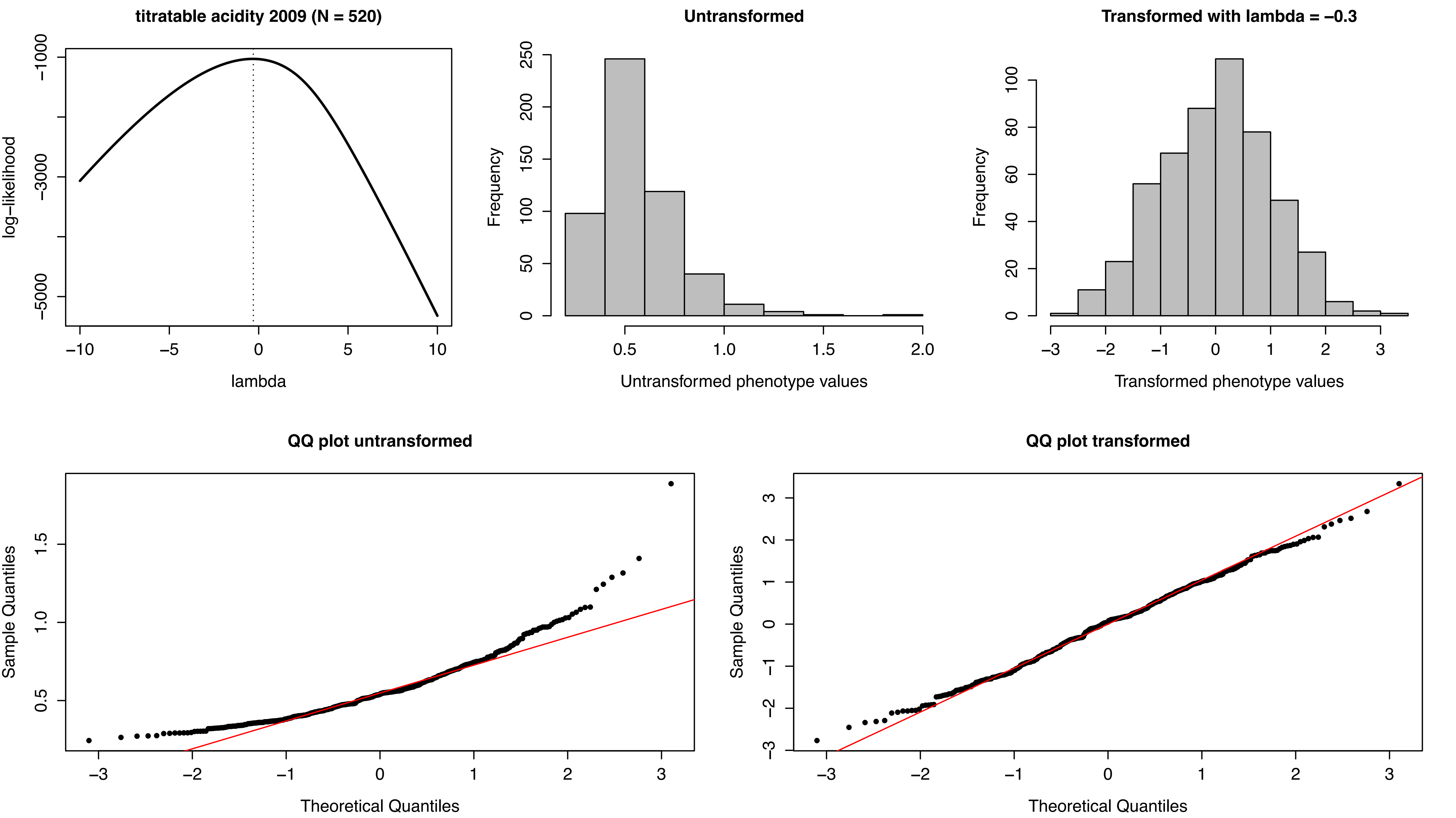
titratable acidity 2009 (N = 520)
log−likelihood
lambda
Untransformed
Frequency
Untransformed phenotype values
Transformed with lambda = −0.3
Frequency
Transformed phenotype values
QQ plot untransformed
Sample Quantiles
Theoretical Quantiles
QQ plot transformed
Sample Quantiles
Theoretical Quantiles

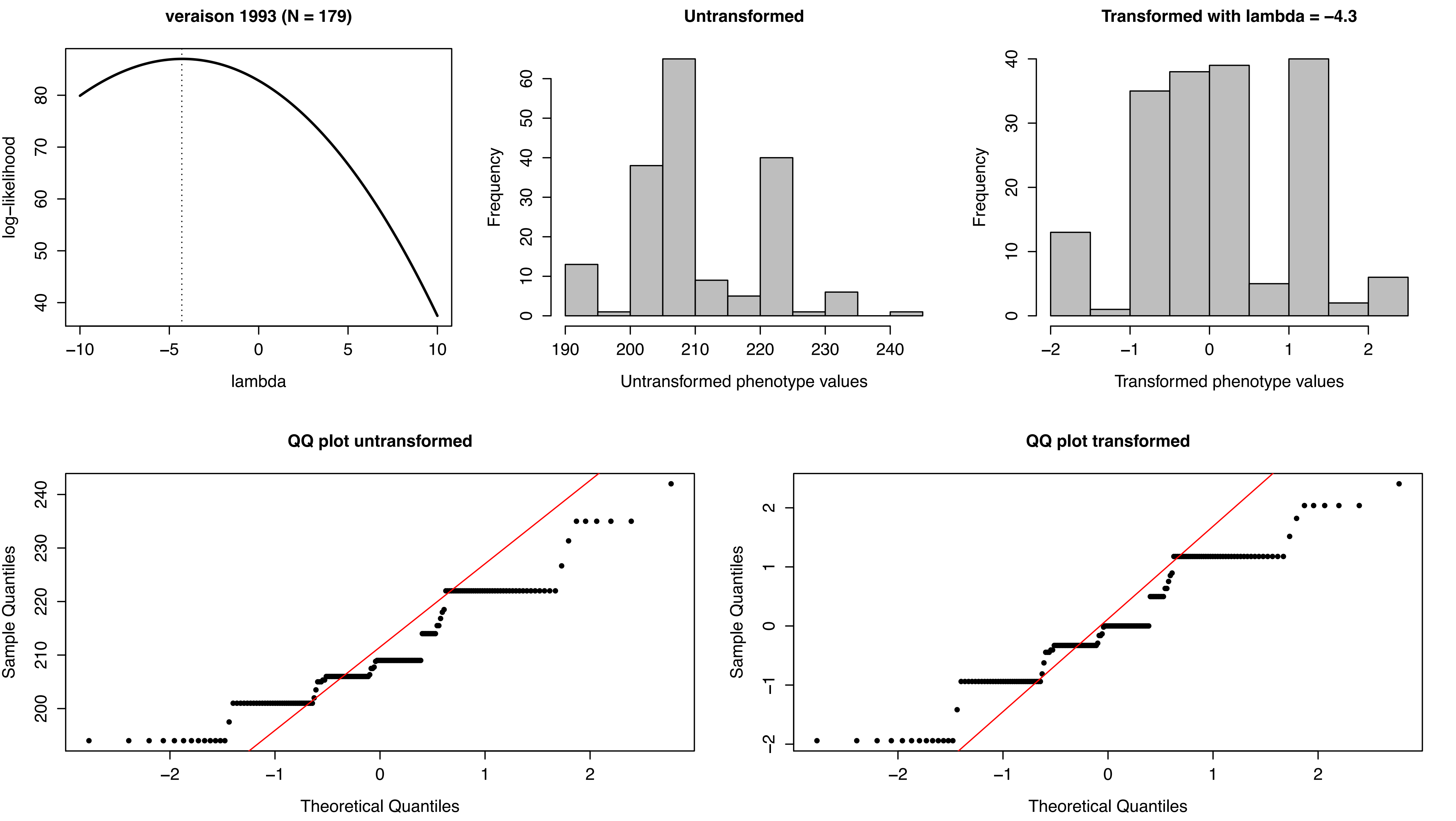
veraison 1993 (N = 179)
log−likelihood
lambda
Untransformed
Frequency
Untransformed phenotype values
Transformed with lambda = −4.3
Frequency
Transformed phenotype values
QQ plot untransformed
Sample Quantiles
Theoretical Quantiles
QQ plot transformed
Sample Quantiles
Theoretical Quantiles
