# Supplementary material for: Patterns of genomic and phenomic diversity in wine and table grapes
Source: Hortic Res. 2017 Aug 2;4:17035–. doi: 10.1038/hortres.2017.35 (PMC5539807; doi:10.1038/hortres.2017.35)
Supplement: Supplementary Figure S2 [file hortres201735-s2.pdf]

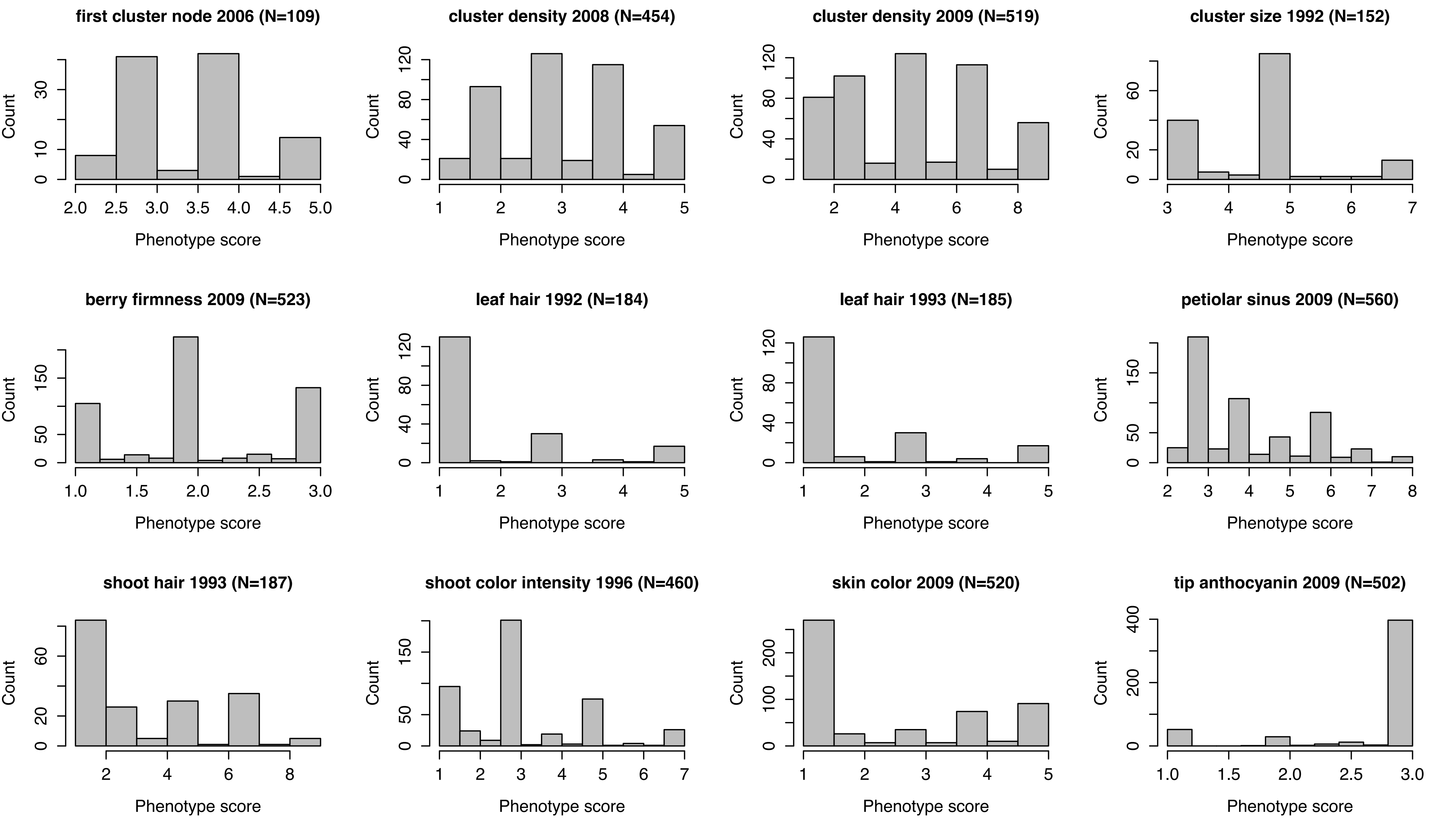
first cluster node 2006 (N=109)
cluster density 2008 (N=454)
cluster density 2009 (N=519)
cluster size 1992 (N=152)
berry firmness 2009 (N=523)
leaf hair 1992 (N=184)
leaf hair 1993 (N=185)
petiolar sinus 2009 (N=560)
shoot hair 1993 (N=187)
shoot color intensity 1996 (N=460)
skin color 2009 (N=520)
tip anthocyanin 2009 (N=502)
Count
Phenotype score
