# Supplementary figures and images for: Patterns of genomic and phenomic diversity in wine and table grapes
Source: Hortic Res. 2017 Aug 2;4:17035–. doi: 10.1038/hortres.2017.35 (PMC5539807; doi:10.1038/hortres.2017.35)

**muscat aroma 2009 (N=519)**

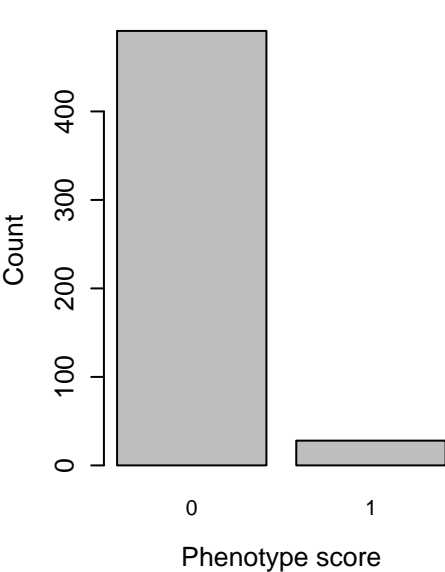

**flower sex 2010 (N=550)**

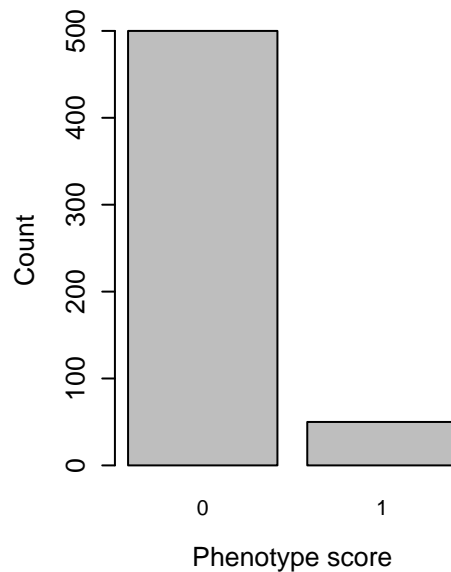

**leaf size 1992 (N=185)**

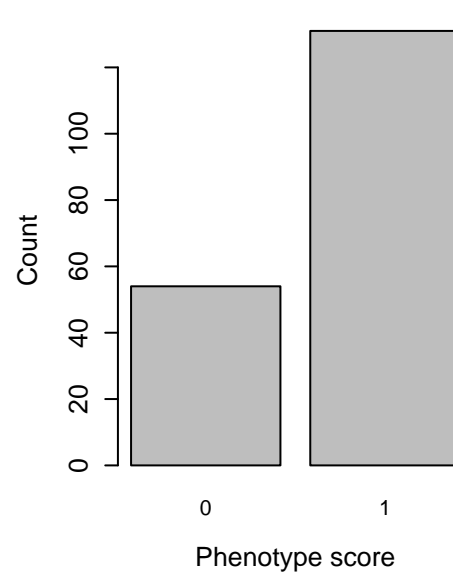

**leaf size 1993 (N=185)**

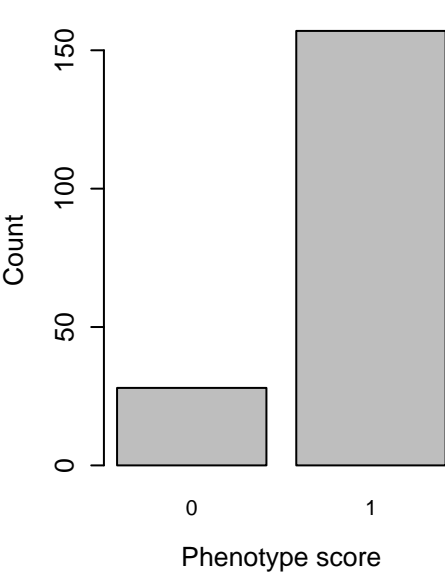

**naked vein 2009 (N=558)**

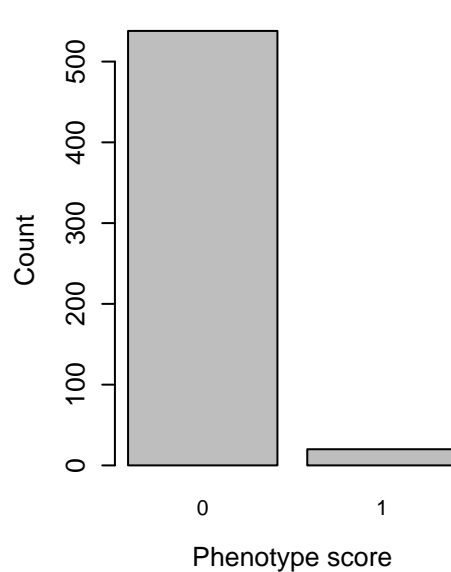

**seedlessness 1993 (N=174)**

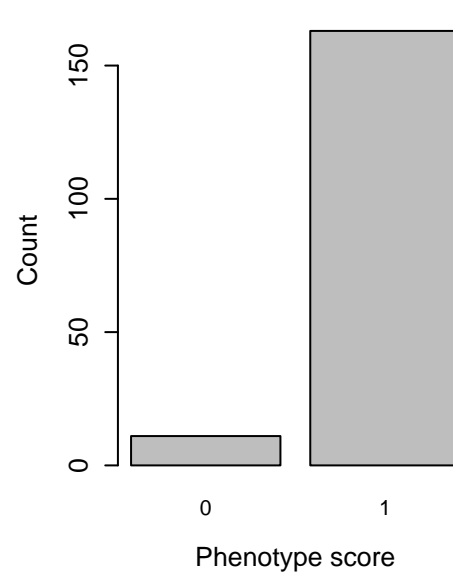

Supplement: Supplementary Figure S3 [file hortres201735-s3.pdf]

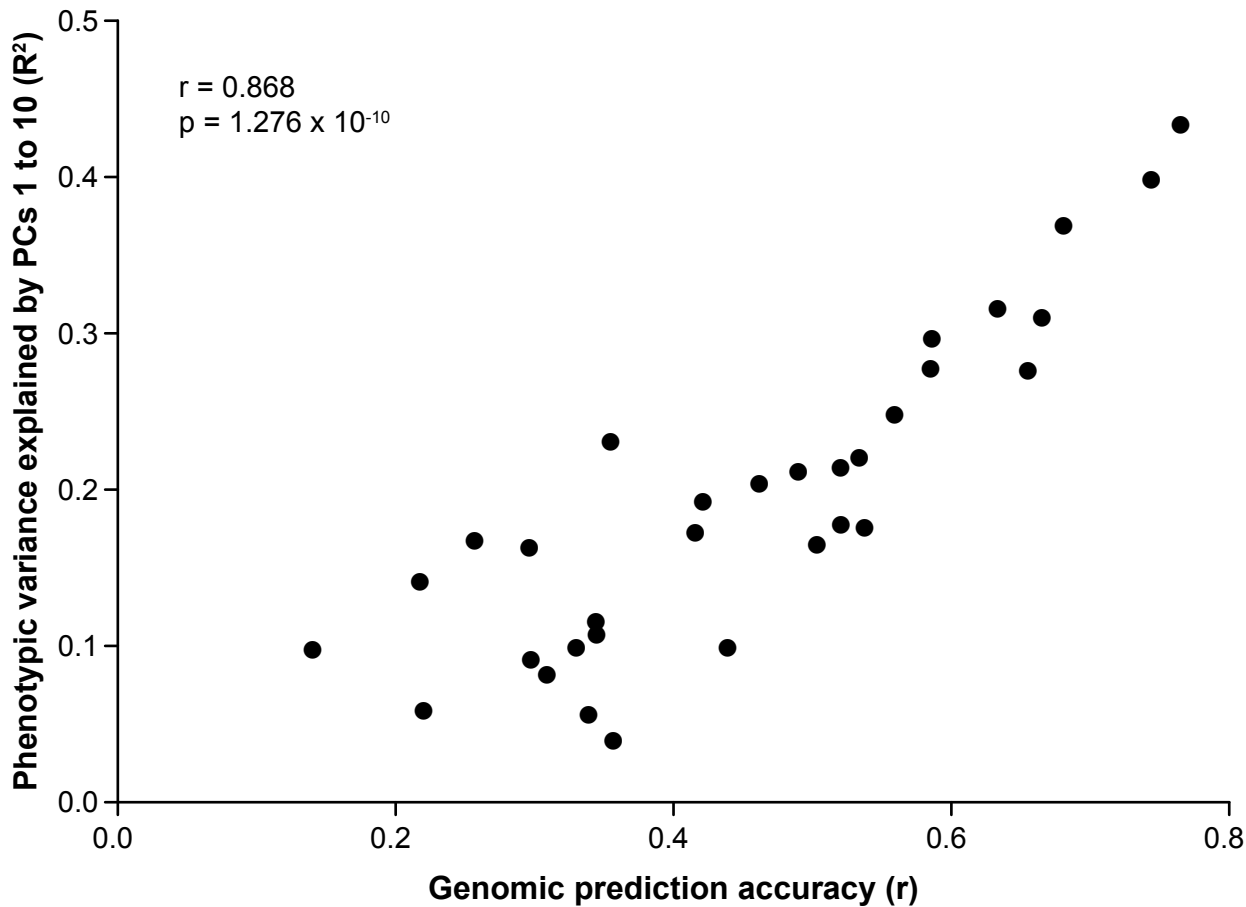

Supplement: Supplementary Figure S4 [file hortres201735-s4.pdf]
