# Supplementary material for: Patterns of genomic and phenomic diversity in wine and table grapes
Source: Hortic Res. 2017 Aug 2;4:17035–. doi: 10.1038/hortres.2017.35 (PMC5539807; doi:10.1038/hortres.2017.35)
Supplement: Supplementary Figure S5 [file hortres201735-s5.pdf]

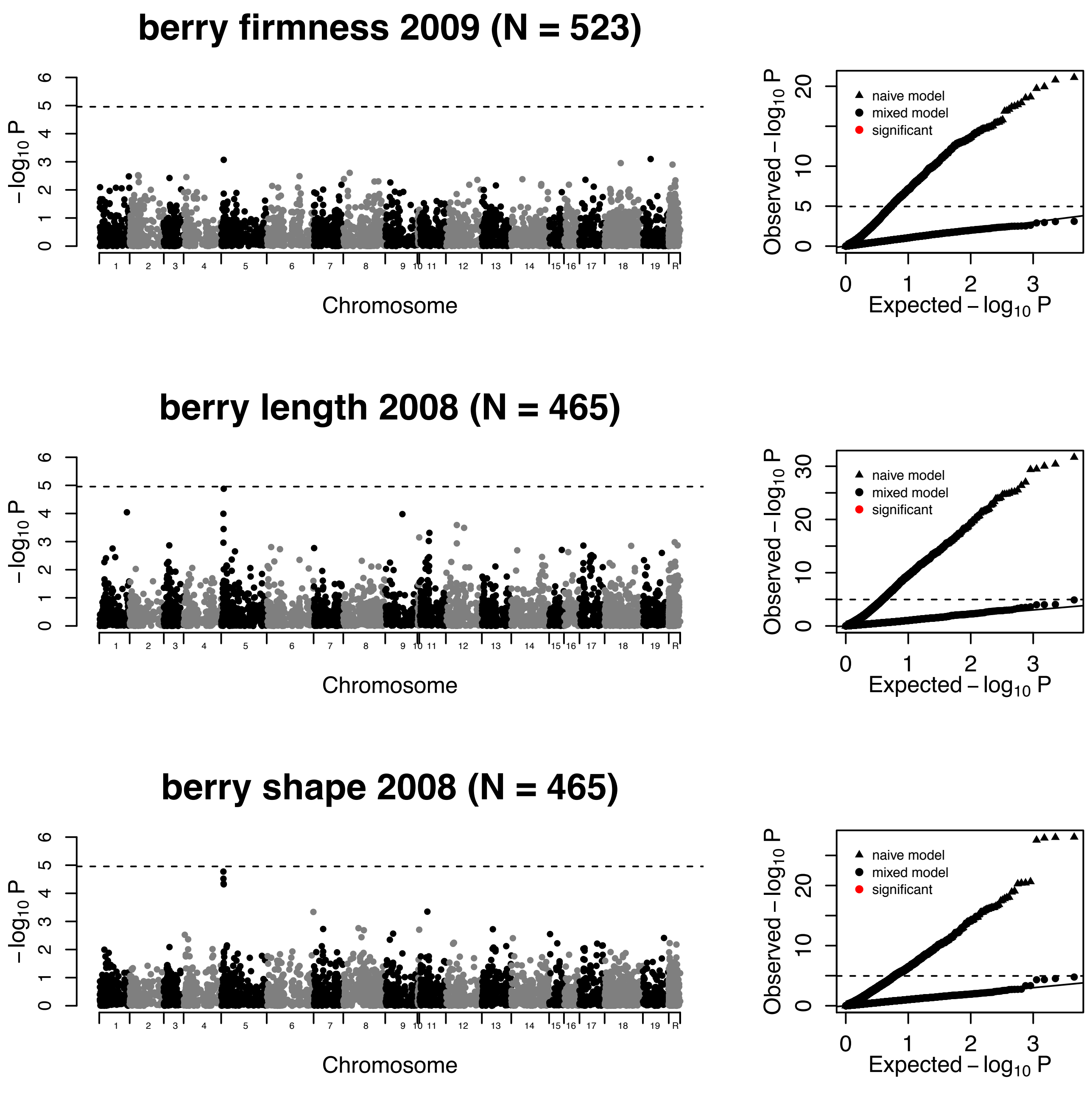

berry firmness 2009 (N = 523)
berry length 2008 (N = 465)
berry shape 2008 (N = 465)
−log10 P
Chromosome
1 2 3 4 5 6 7 8 9 10 11 12 13 14 15 16 17 18 19 R
Observed − log10 P
Expected − log10 P
naive model
mixed model
significant

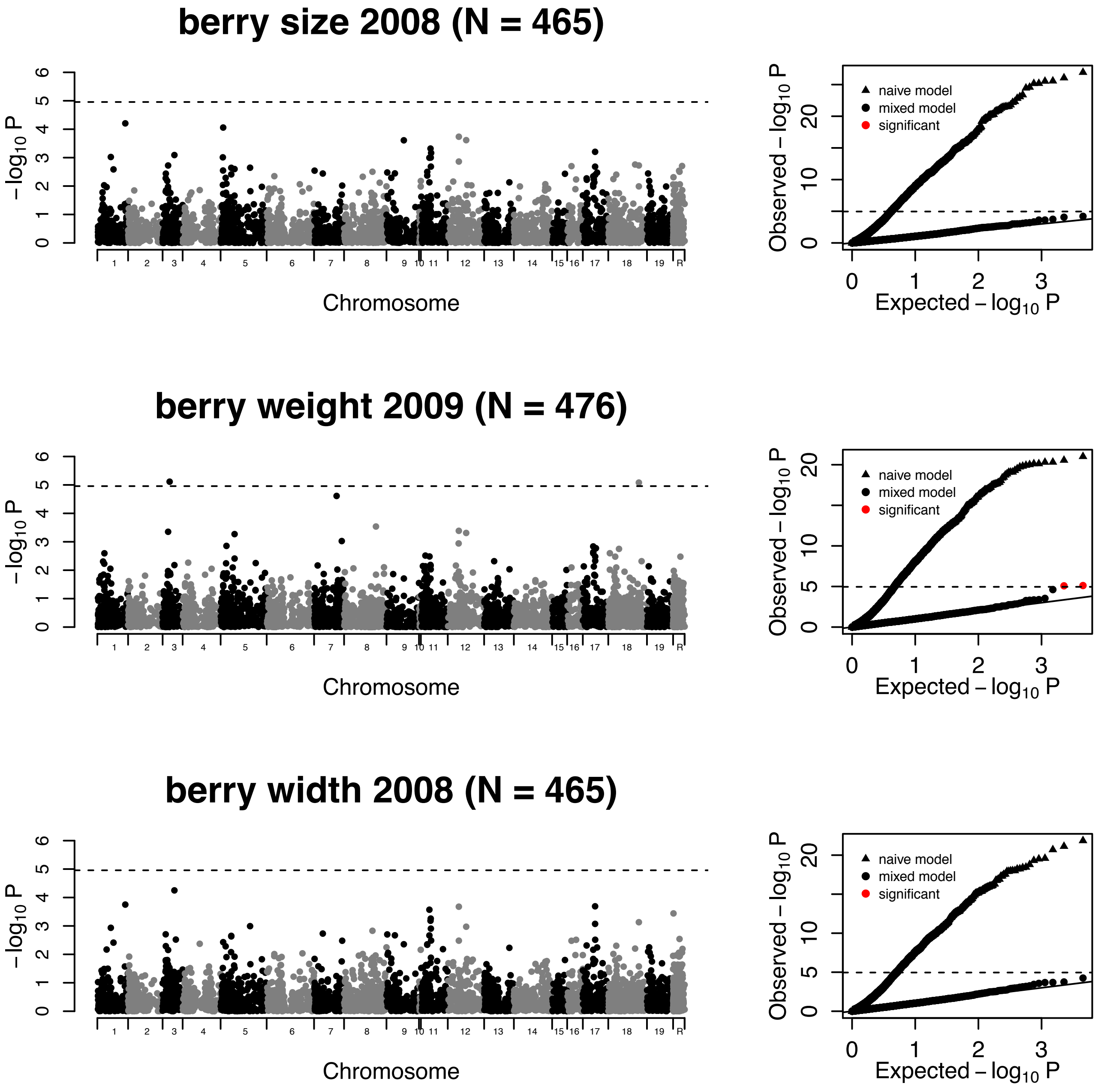

berry size 2008 (N = 465)
berry weight 2009 (N = 476)
berry width 2008 (N = 465)
−log10 P
Chromosome
1 2 3 4 5 6 7 8 9 10 11 12 13 14 15 16 17 18 19 R
naive model
mixed model
significant
Observed − log10 P
Expected − log10 P

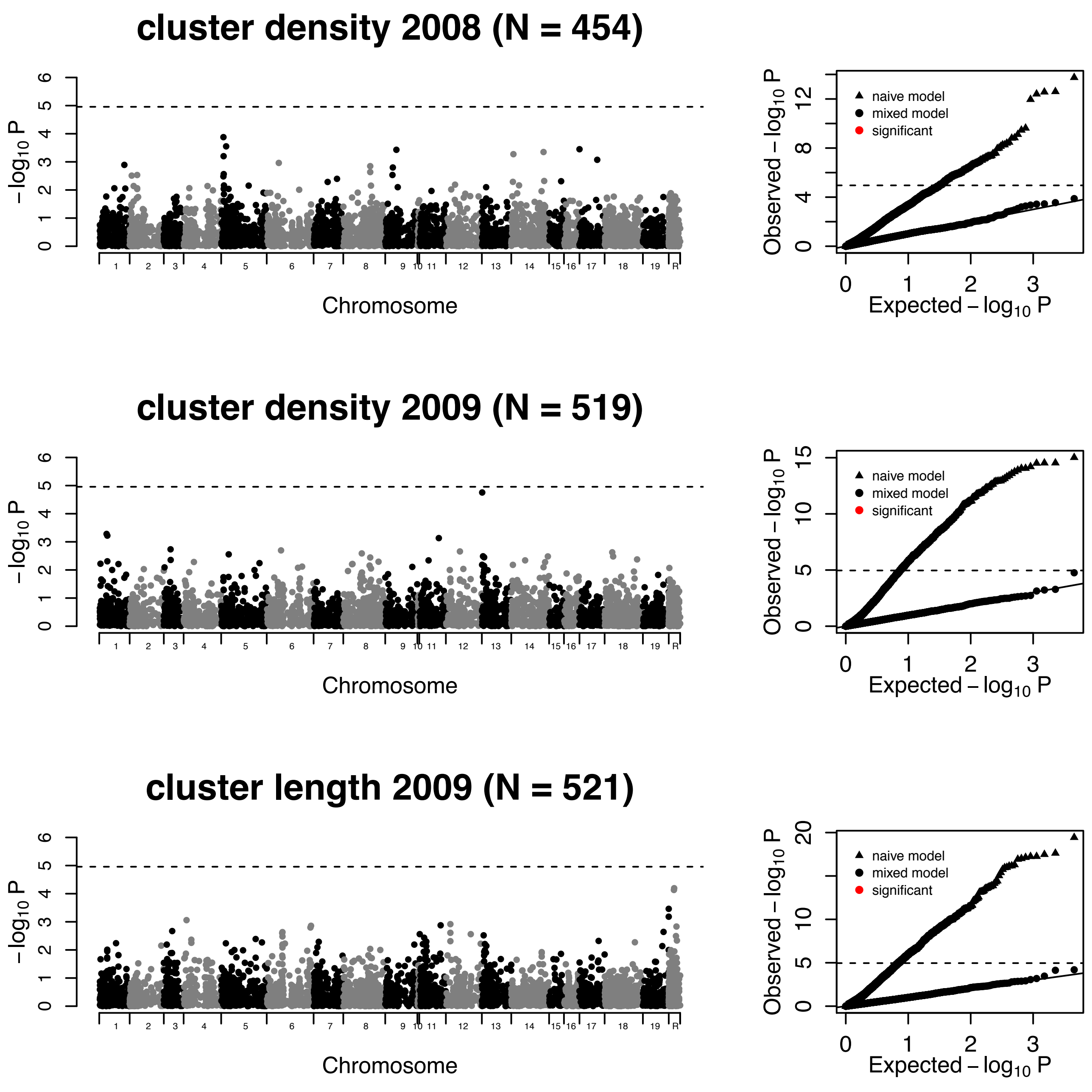
cluster density 2008 (N = 454)
cluster density 2009 (N = 519)
cluster length 2009 (N = 521)
-log10 P
Chromosome
1 2 3 4 5 6 7 8 9 10 11 12 13 14 15 16 17 18 19 R
Observed - log10 P
Expected - log10 P
naive model
mixed model
significant

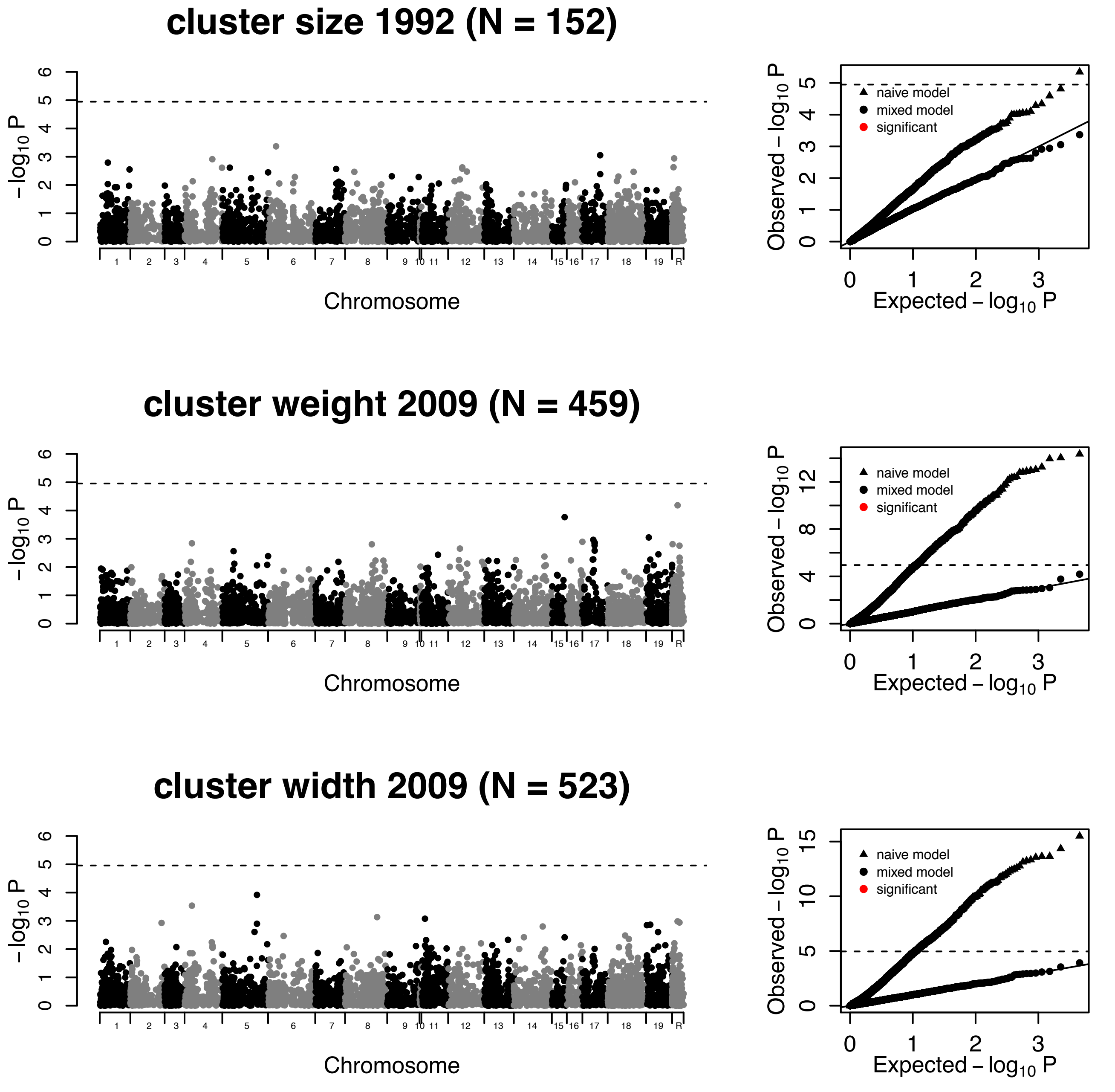
cluster size 1992 (N = 152)
cluster weight 2009 (N = 459)
cluster width 2009 (N = 523)
−log10 P
Chromosome
1 2 3 4 5 6 7 8 9 10 11 12 13 14 15 16 17 18 19 R
Observed − log10 P
Expected − log10 P
naive model
mixed model
significant

field brix 1993 (N = 184)

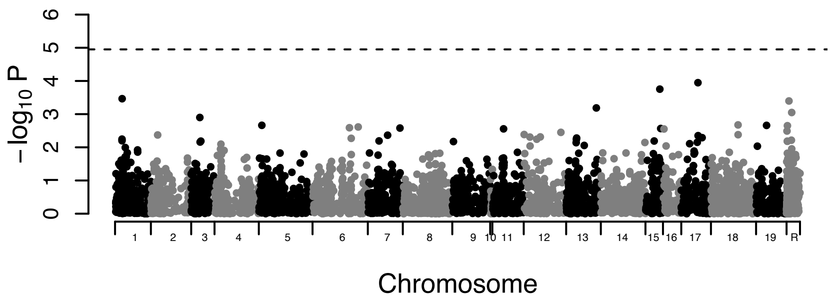

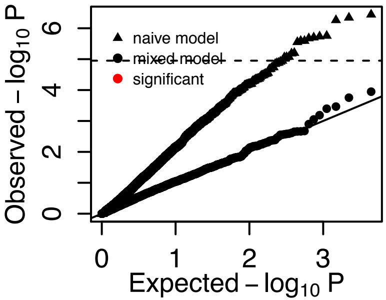

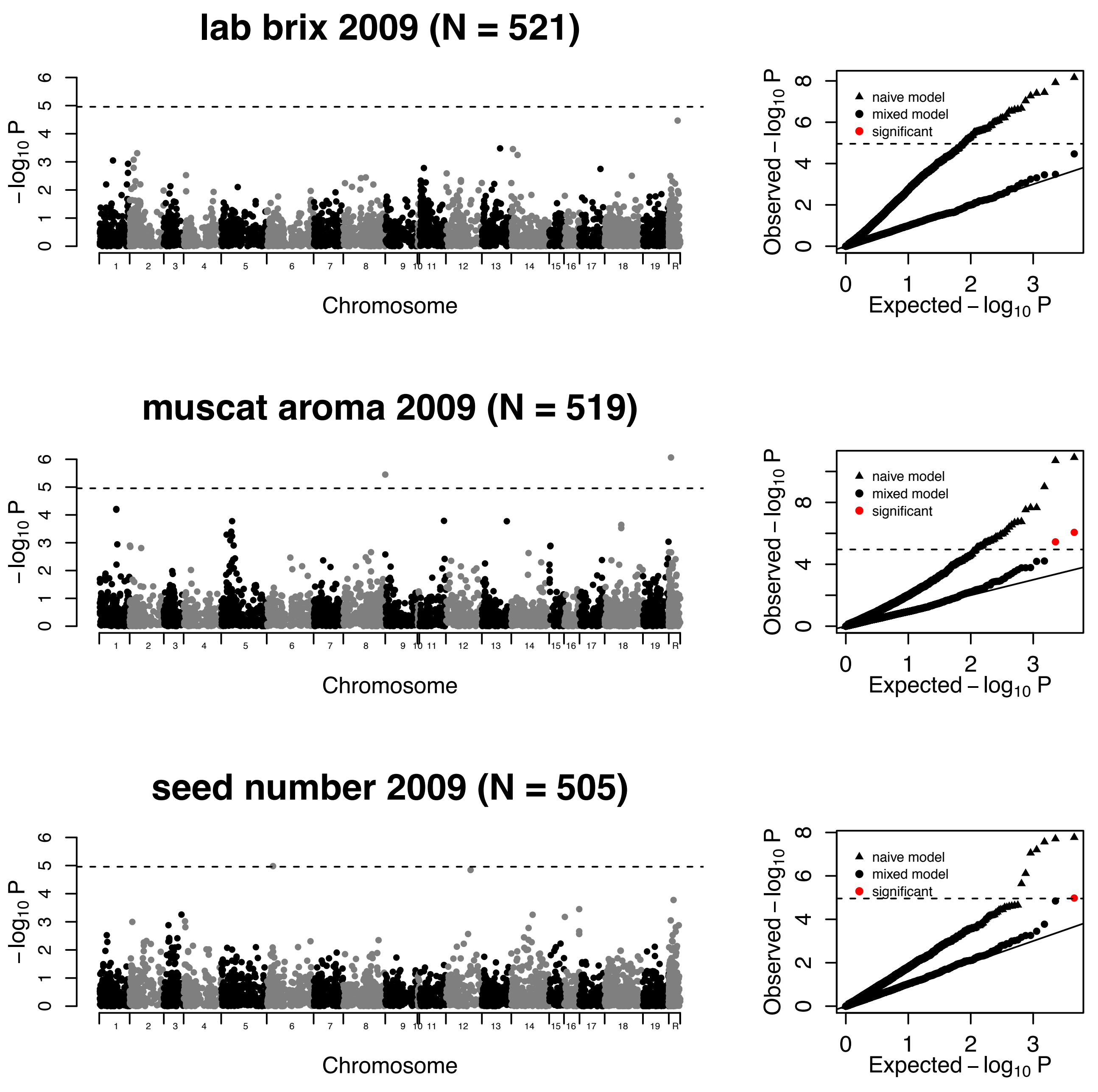
lab brix 2009 (N = 521)
muscat aroma 2009 (N = 519)
seed number 2009 (N = 505)
−log10 P
Chromosome
1 2 3 4 5 6 7 8 9 10 11 12 13 14 15 16 17 18 19 R
naive model
mixed model
significant
Observed − log10 P
Expected − log10 P

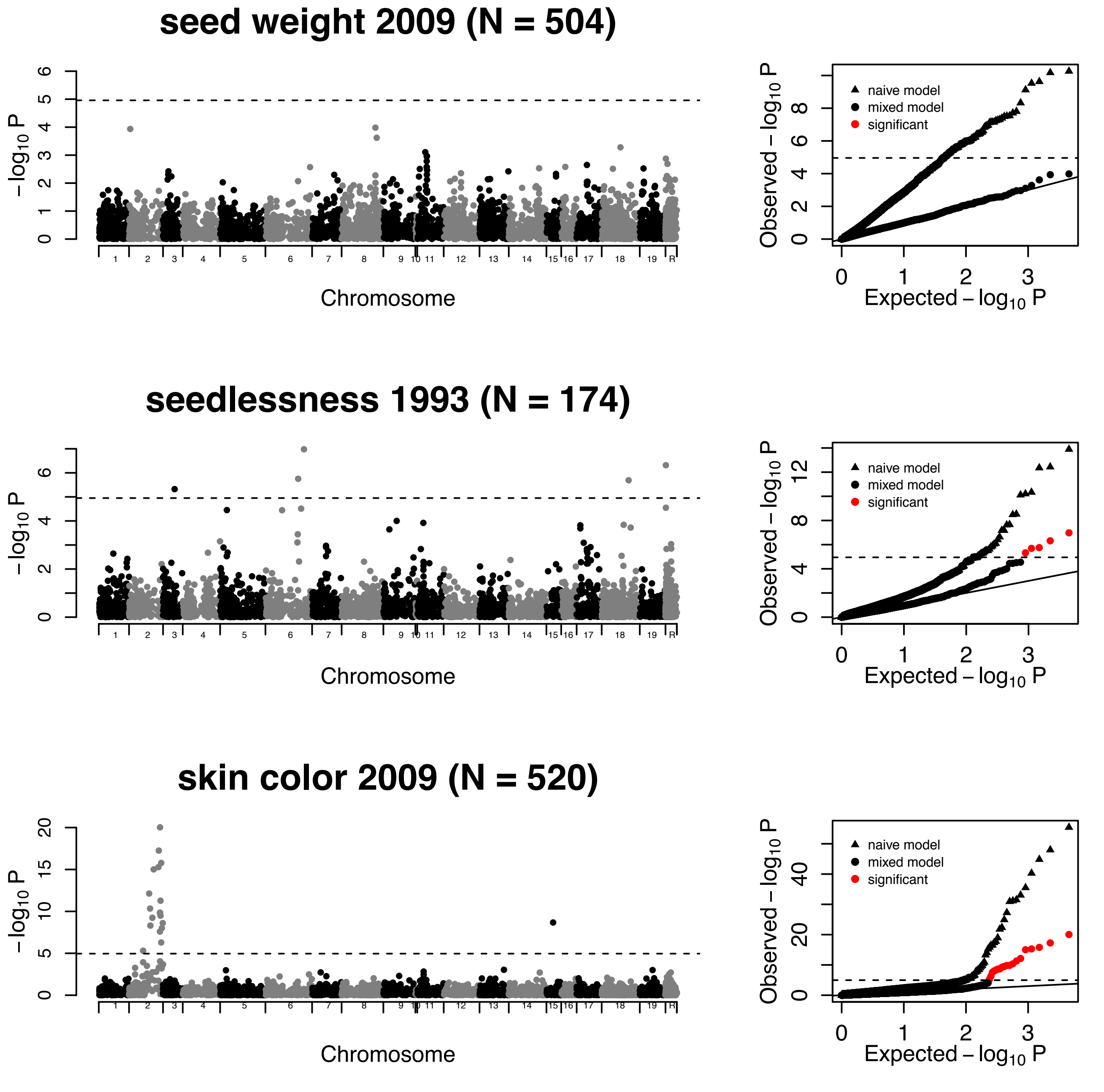

seed weight 2009 (N = 504)
−log10 P
Chromosome
1 2 3 4 5 6 7 8 9 10 11 12 13 14 15 16 17 18 19 R
Observed − log10 P
Expected − log10 P
naive model
mixed model
significant
seedlessness 1993 (N = 174)
−log10 P
Chromosome
Observed − log10 P
Expected − log10 P
naive model
mixed model
significant
skin color 2009 (N = 520)
−log10 P
Chromosome
Observed − log10 P
Expected − log10 P
naive model
mixed model
significant

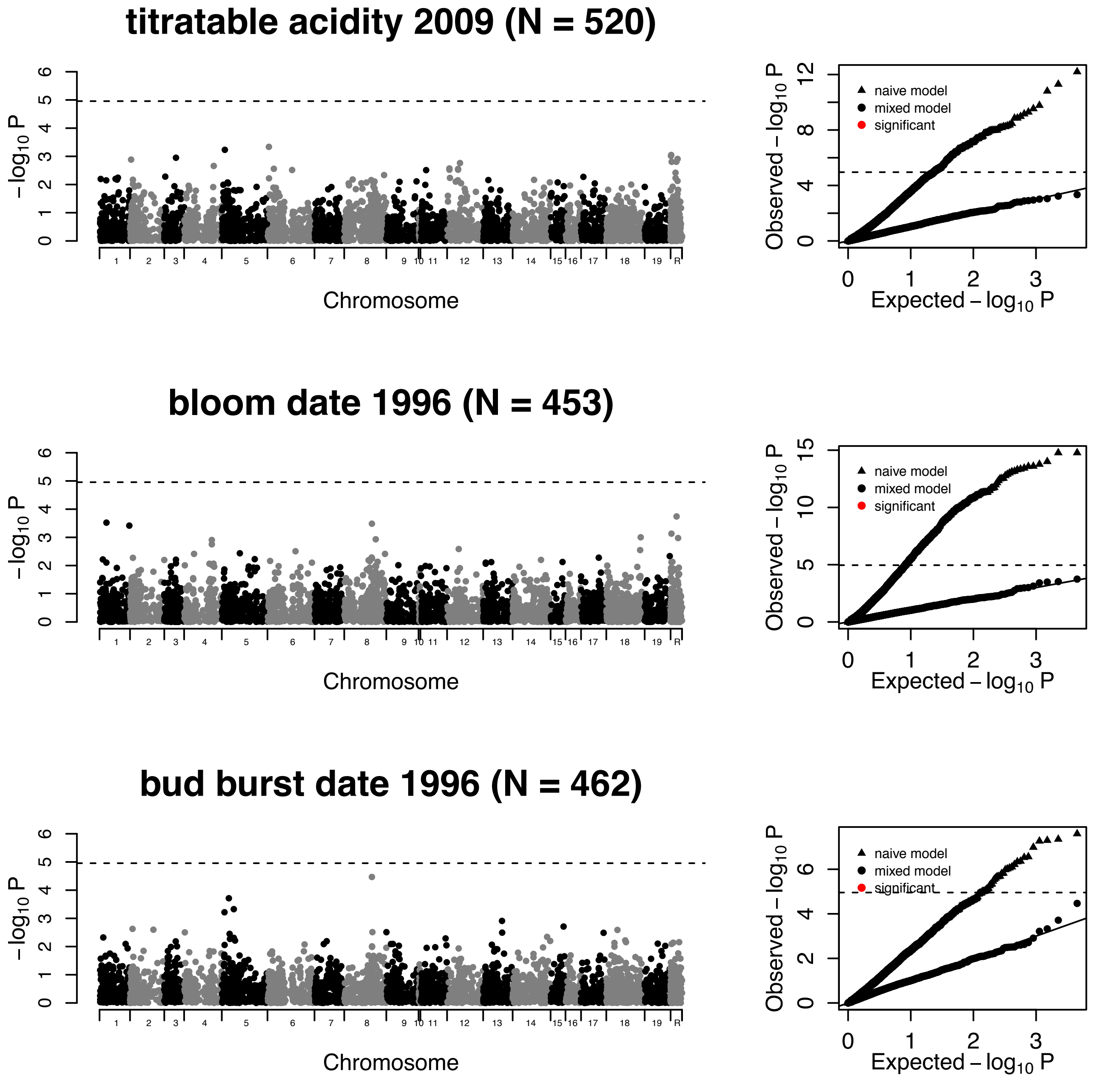

titratable acidity 2009 (N = 520)
bloom date 1996 (N = 453)
bud burst date 1996 (N = 462)
−log10 P
Chromosome
1 2 3 4 5 6 7 8 9 10 11 12 13 14 15 16 17 18 19 R
Observed − log10 P
Expected − log10 P
naive model
mixed model
significant

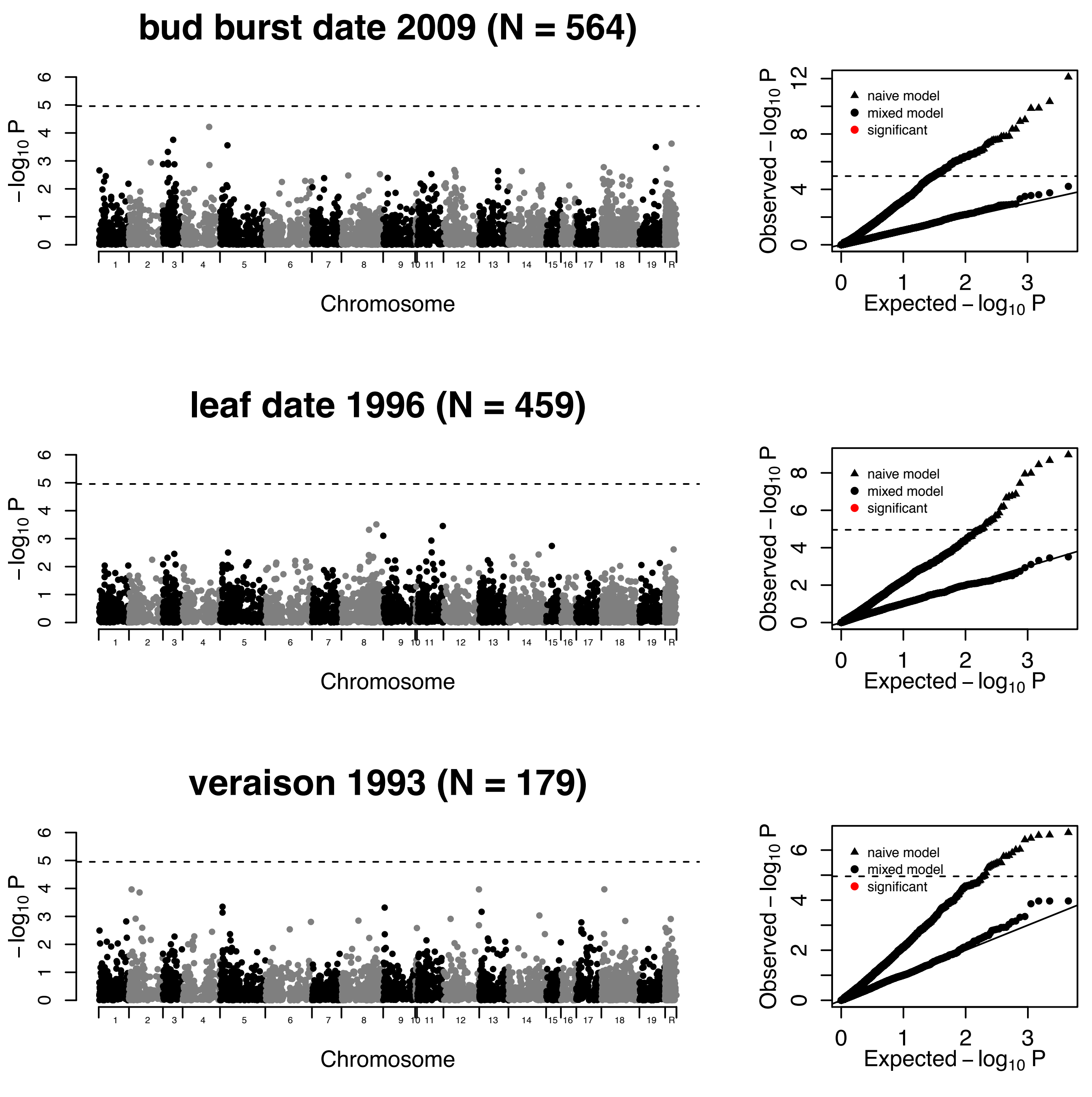

bud burst date 2009 (N = 564)
leaf date 1996 (N = 459)
veraison 1993 (N = 179)
$-\log_{10} P$
Chromosome
1 2 3 4 5 6 7 8 9 10 11 12 13 14 15 16 17 18 19 R
Observed $-\log_{10}$ P
Expected $-\log_{10}$ P
naive model
mixed model
significant

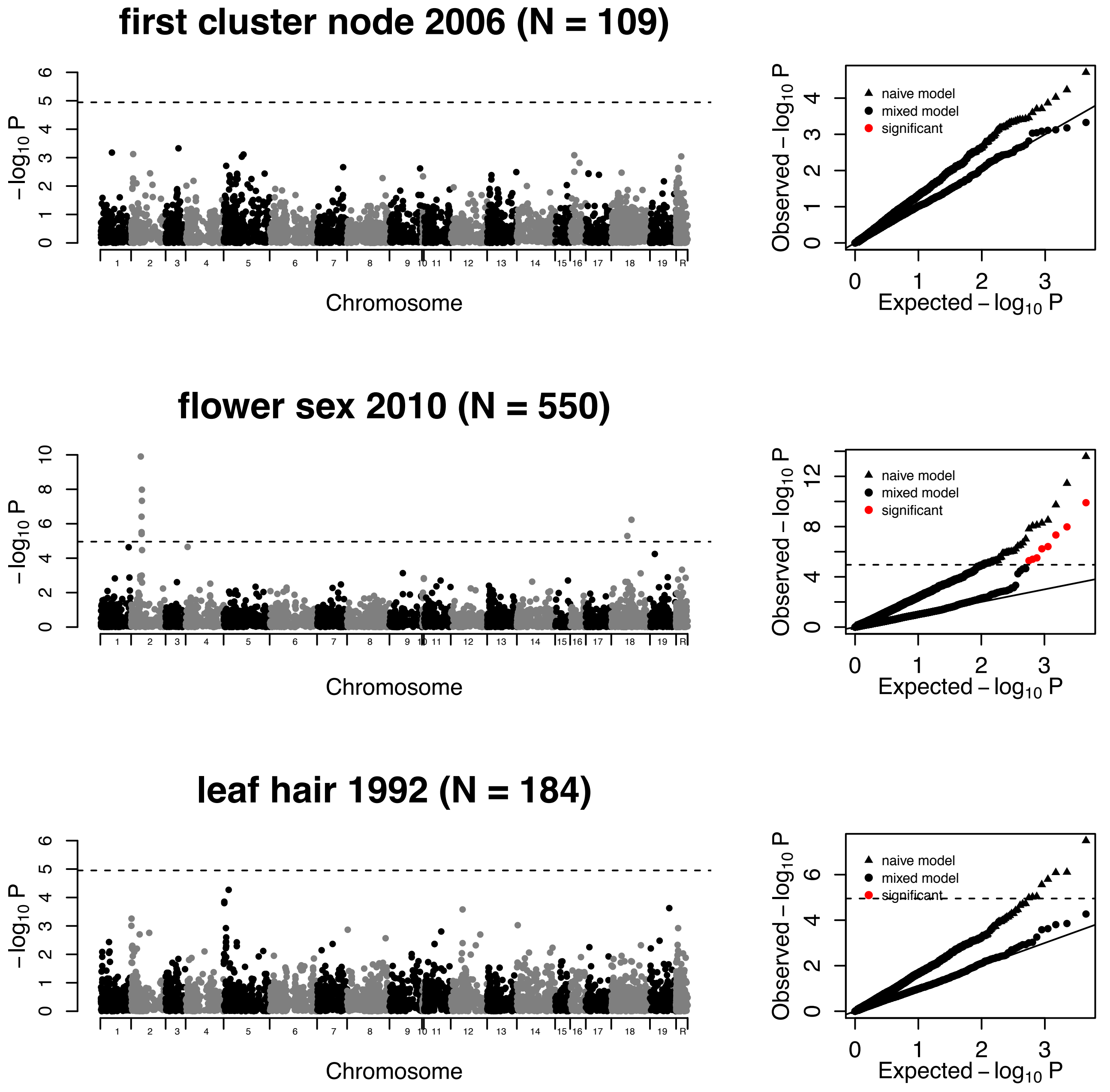
first cluster node 2006 (N = 109)
flower sex 2010 (N = 550)
leaf hair 1992 (N = 184)
$-\log_{10}$ P
Chromosome
1 2 3 4 5 6 7 8 9 10 11 12 13 14 15 16 17 18 19 R
Observed $-\log_{10}$ P
Expected $-\log_{10}$ P
naive model
mixed model
significant

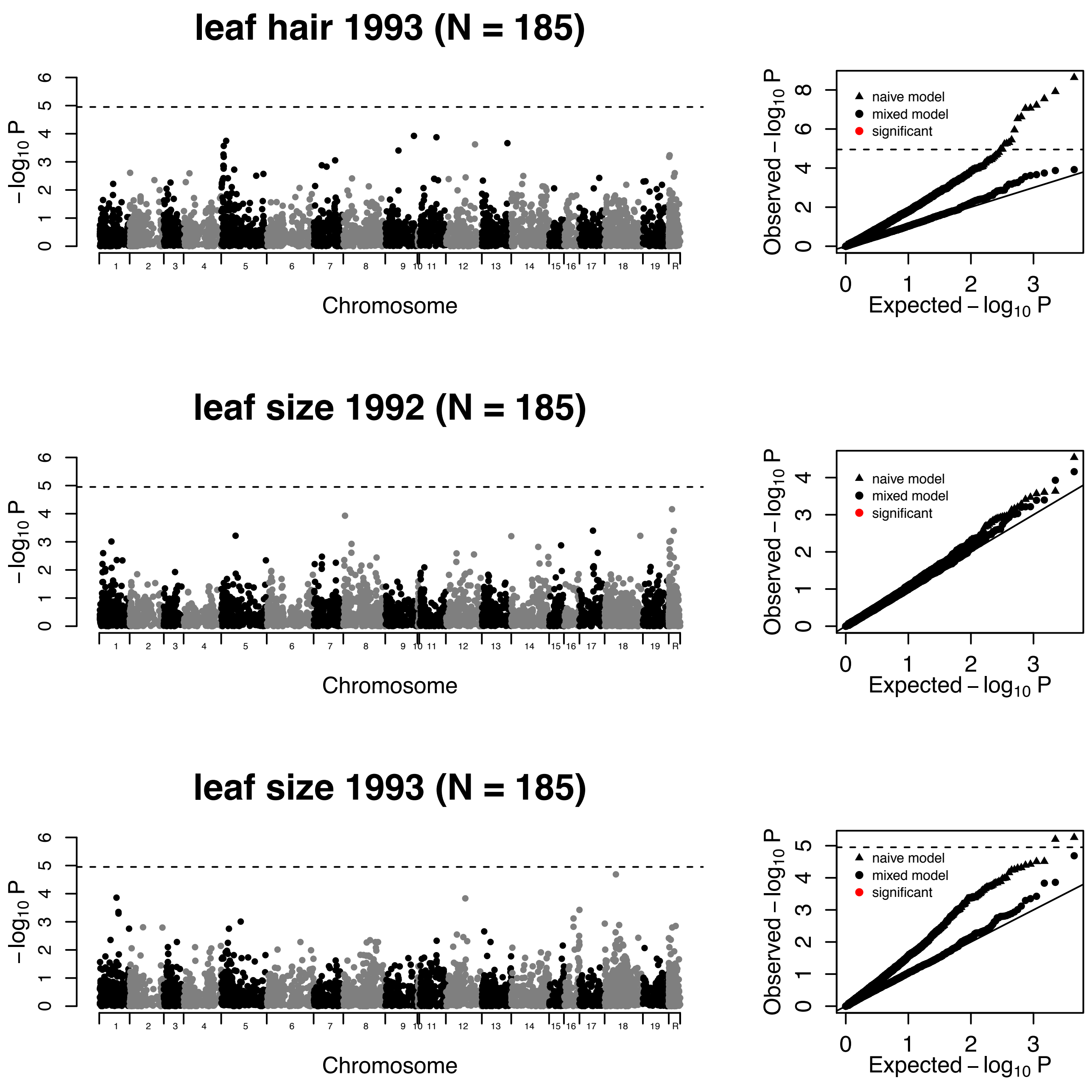
leaf hair 1993 (N = 185)
−log10 P
Chromosome
1 2 3 4 5 6 7 8 9 10 11 12 13 14 15 16 17 18 19 R
naive model
mixed model
significant
Observed −log10 P
Expected −log10 P
leaf size 1992 (N = 185)
−log10 P
Chromosome
1 2 3 4 5 6 7 8 9 10 11 12 13 14 15 16 17 18 19 R
naive model
mixed model
significant
Observed −log10 P
Expected −log10 P
leaf size 1993 (N = 185)
−log10 P
Chromosome
1 2 3 4 5 6 7 8 9 10 11 12 13 14 15 16 17 18 19 R
naive model
mixed model
significant
Observed −log10 P
Expected −log10 P

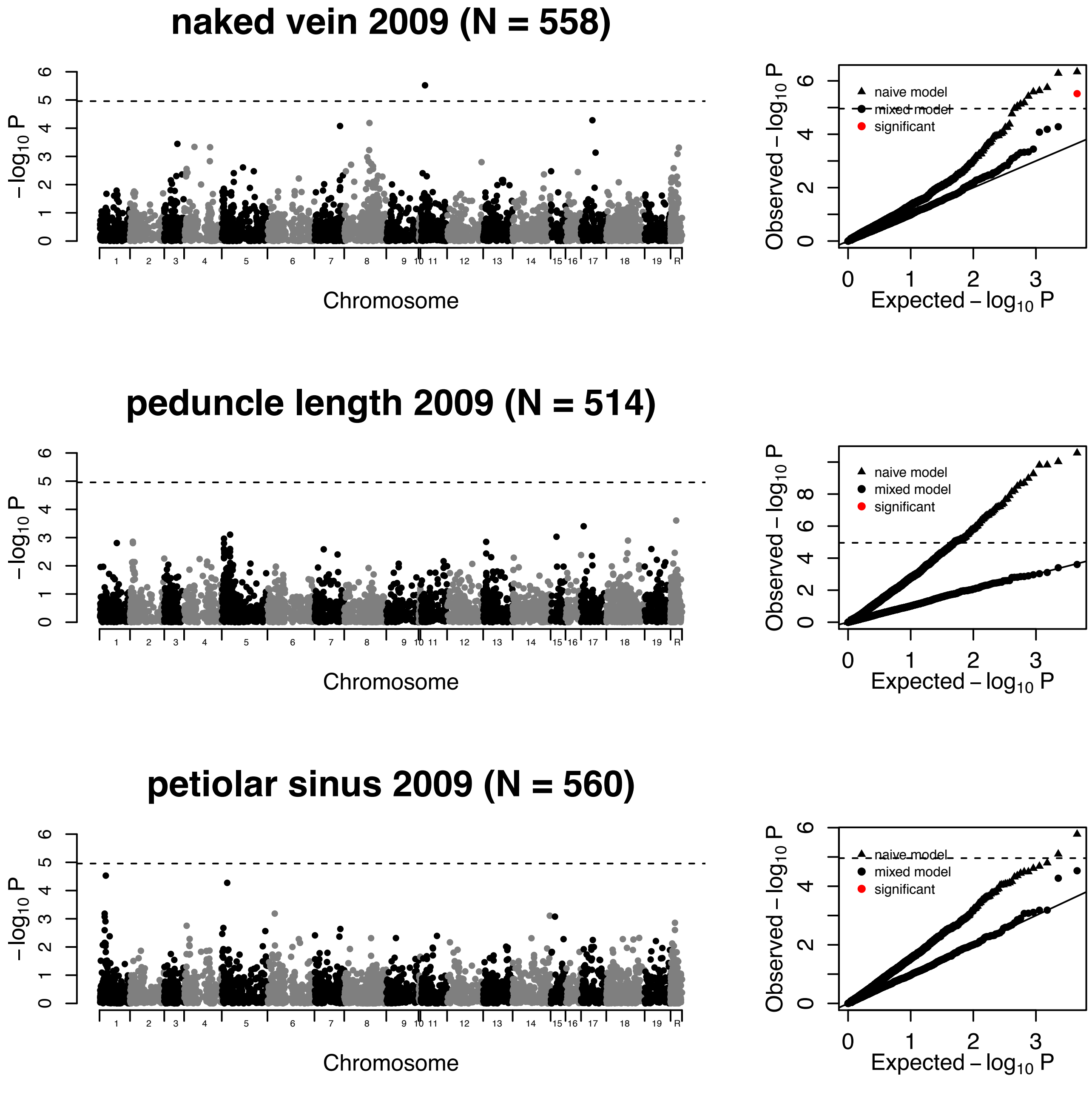

naked vein 2009 (N = 558)
peduncle length 2009 (N = 514)
petiolar sinus 2009 (N = 560)
−log10 P
Chromosome
1 2 3 4 5 6 7 8 9 10 11 12 13 14 15 16 17 18 19 R
Observed − log10 P
Expected − log10 P
naive model
mixed model
significant

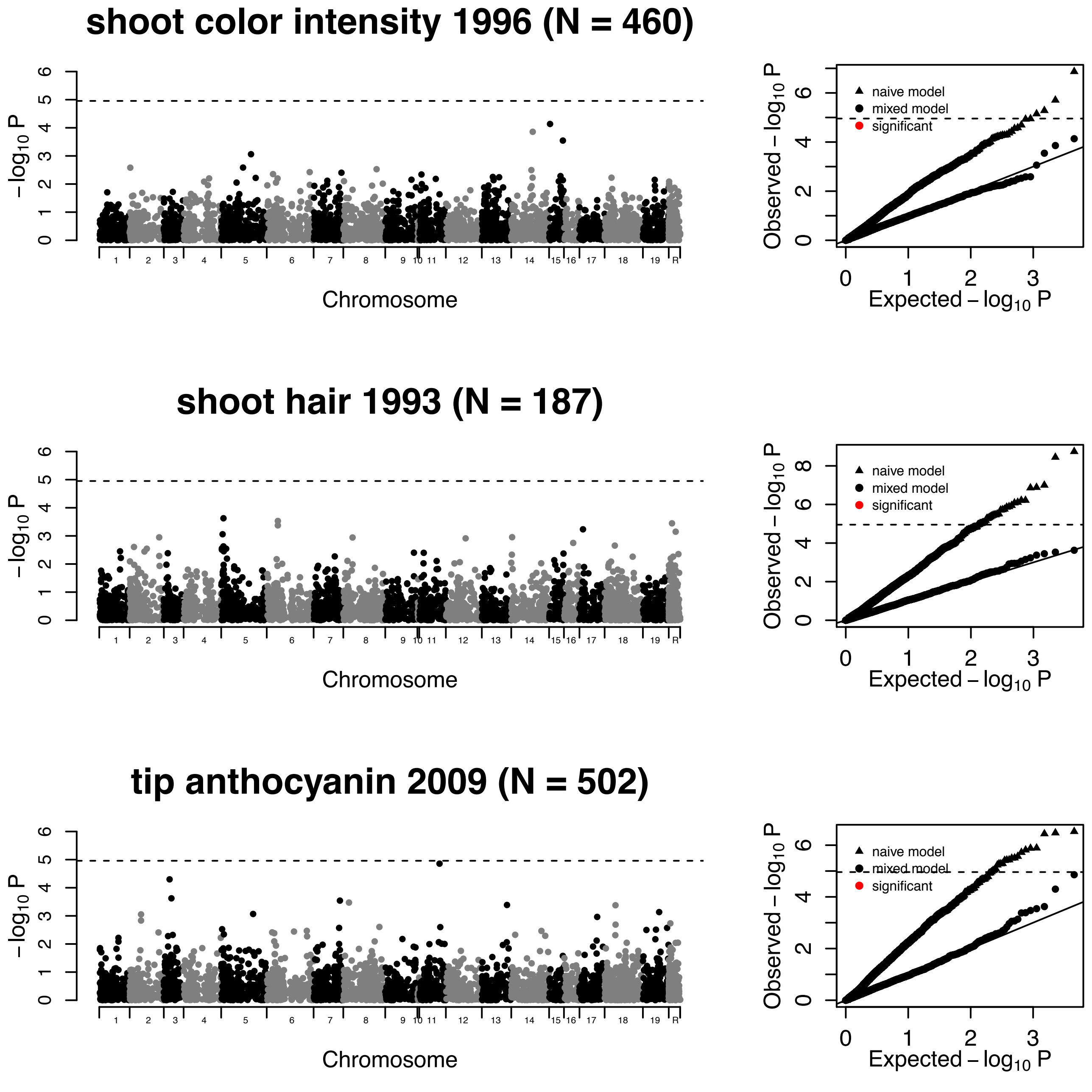
shoot color intensity 1996 (N = 460)
shoot hair 1993 (N = 187)
tip anthocyanin 2009 (N = 502)
−log10 P
Chromosome
1 2 3 4 5 6 7 8 9 10 11 12 13 14 15 16 17 18 19 R
Observed − log10 P
Expected − log10 P
naive model
mixed model
significant
